# The development of a core outcome set for studies of pregnant women with multimorbidity

**DOI:** 10.1186/s12916-023-03013-3

**Published:** 2023-08-21

**Authors:** Siang Ing Lee, Stephanie Hanley, Zoe Vowles, Rachel Plachcinski, Ngawai Moss, Megha Singh, Chris Gale, Adeniyi Francis Fagbamigbe, Amaya Azcoaga-Lorenzo, Anuradhaa Subramanian, Beck Taylor, Catherine Nelson-Piercy, Christine Damase-Michel, Christopher Yau, Colin McCowan, Dermot O’Reilly, Gillian Santorelli, Helen Dolk, Holly Hope, Katherine Phillips, Kathryn M. Abel, Kelly-Ann Eastwood, Lisa Kent, Louise Locock, Maria Loane, Mohamed Mhereeg, Peter Brocklehurst, Sharon McCann, Sinead Brophy, Steven Wambua, Sudasing Pathirannehelage Buddhika Hemali Sudasinghe, Shakila Thangaratinam, Krishnarajah Nirantharakumar, Mairead Black, on behalf of the MuM-PreDiCT Group

**Affiliations:** 1 Institute of Applied Health Research, University of Birmingham, Birmingham, UK; 2 Guy’s and St. Thomas’ NHS Foundation Trust, London, UK; 3 Patient and public representative, London, UK; 4 Neonatal Medicine, School of Public Health, Faculty of Medicine, Imperial College London, London, UK; 5 Division of Population and Behavioural Sciences, School of Medicine, University of St Andrews, St Andrews, UK; 6 Department of Epidemiology and Medical Statistics, College of Medicine, University of Ibadan, Ibadan, Nigeria; 7 Hospital Rey Juan Carlos, Instituto de Investigación Sanitaria Fundación Jimenez Diaz, Madrid, Spain; 8 Medical and Clinical Pharmacology, School of Medicine, Université Toulouse III, Toulouse, France; 9 Center for Epidemiology and Research in Population Health (CERPOP), INSERM, Toulouse, France; 10 Nuffield Department of Women’s and Reproductive Health, University of Oxford, Oxford, UK; 11 Health Data Research UK, London, UK; 12 Centre for Public Health, Queen’s University of Belfast, Belfast, UK; 13 Bradford Institute for Health Research, Bradford, UK; 14 Centre for Maternal, Fetal and Infant Research, Ulster University, Belfast, UK; 15 Centre for Women’s Mental Health, Faculty of Biology Medicine & Health, The University of Manchester, Manchester, UK; 16 Greater Manchester Mental Health NHS Foundation Trust, Manchester, UK; 17 St Michael’s Hospital, University Hospitals Bristol NHS Foundation Trust, Bristol, UK; 18 Health Services Research Unit, Health Sciences Building, Foresterhill, University of Aberdeen, Aberdeen, UK; 19 The Institute of Nursing and Health Research, Ulster University, Newtownabbey, UK; 20 Data Science, Medical School, Swansea University, Swansea, UK; 21 WHO Collaborating Centre for Global Women’s Health, Institute of Metabolism and Systems Research, University of Birmingham, Birmingham, UK; 22 Department of Obstetrics and Gynaecology, Birmingham Women’s and Children’s NHS Foundation Trust, Birmingham, UK; 23 Aberdeen Centre for Women’s Health Research, School of Medicine, Medical Science and Nutrition, University of Aberdeen, Aberdeen, UK

**Keywords:** Multimorbidity, Multiple chronic conditions, Multiple long-term conditions, Pregnancy, Maternity, Outcome, Core outcome set

## Abstract

**Background:**

Heterogeneity in reported outcomes can limit the synthesis of research evidence. A core outcome set informs what outcomes are important and should be measured as a minimum in all future studies. We report the development of a core outcome set applicable to observational and interventional studies of pregnant women with multimorbidity.

**Methods:**

We developed the core outcome set in four stages: (i) a systematic literature search, (ii) three focus groups with UK stakeholders, (iii) two rounds of Delphi surveys with international stakeholders and (iv) two international virtual consensus meetings. Stakeholders included women with multimorbidity and experience of pregnancy in the last 5 years, or are planning a pregnancy, their partners, health or social care professionals and researchers. Study adverts were shared through stakeholder charities and organisations.

**Results:**

Twenty-six studies were included in the systematic literature search (2017 to 2021) reporting 185 outcomes. Thematic analysis of the focus groups added a further 28 outcomes. Two hundred and nine stakeholders completed the first Delphi survey. One hundred and sixteen stakeholders completed the second Delphi survey where 45 outcomes reached *Consensus In* (≥70% of all participants rating an outcome as *Critically Important*). Thirteen stakeholders reviewed 15 *Borderline* outcomes in the first consensus meeting and included seven additional outcomes. Seventeen stakeholders reviewed these 52 outcomes in a second consensus meeting, the threshold was ≥80% of all participants voting for inclusion. The final core outcome set included 11 outcomes. The five maternal outcomes were as follows: maternal death, severe maternal morbidity, change in existing long-term conditions (physical and mental), quality and experience of care and development of new mental health conditions. The six child outcomes were as follows: survival of baby, gestational age at birth, neurodevelopmental conditions/impairment, quality of life, birth weight and separation of baby from mother for health care needs.

**Conclusions:**

Multimorbidity in pregnancy is a new and complex clinical research area. Following a rigorous process, this complexity was meaningfully reduced to a core outcome set that balances the views of a diverse stakeholder group.

**Supplementary Information:**

The online version contains supplementary material available at 10.1186/s12916-023-03013-3.

## Background

One in five pregnant women in the United Kingdom (UK) has multiple, pre-existing long-term physical or mental health conditions (termed ‘multimorbidity’ hereafter) [1]. Polypharmacy is prevalent in pregnant women with multimorbidity as they may have to manage their health conditions with multiple medication [2]. Recent studies have demonstrated that maternal multimorbidity is associated with adverse outcomes such as hypertensive disorders of pregnancy, utilisation of acute health services during the perinatal period, preterm birth, severe maternal morbidity and maternal mortality [3–5]. However, this evidence is sparse and the population is under-researched [3]. The impact of polypharmacy on the pregnancy, the women and her child is also unclear.

Research priorities for multimorbidity in pregnancy include understanding the long-term consequences for mother and child and developing new interventions and models of care [6]. Both observational and interventional studies are needed to provide information that can help women with multimorbidity make informed decisions with their clinicians, and to develop interventions that will improve outcomes for mother and child. For instance, longitudinal observational studies are crucial to providing evidence on children’s long-term outcomes.

As research in this field gains momentum globally [3, 7, 8], a core outcome set is needed to avoid heterogeneity of reported outcomes, which can limit the synthesis of research and its usability [9]. A core outcome set informs what outcomes are important and should be reported as a minimum in all future studies for a particular health condition [10]. To ensure its relevance, core outcome sets are developed through consensus-setting methods with stakeholders including people living with the health conditions, health and social care professionals and researchers [10].

There are currently limited core outcome sets available for long-term conditions in pregnancy; examples include core outcome sets for epilepsy [11], diabetes [12], heart conditions [13] and rheumatological conditions in pregnancy [14]. Core outcome sets for pregnancies in general [15, 16] and for medication safety in pregnancy [17] do not have outcomes reflecting challenges specific to women with multimorbidity, such as the impact of pregnancy on their long-term conditions. Conversely, the core outcome set for multimorbidity [18] does not have pregnancy outcomes. To address this gap, and to guide future studies in this field, a core outcome set specific for pregnant women with multimorbidity is needed. This paper reports the development of a core outcome set for studies of pregnant women with multimorbidity.

## Methods

### Inclusivity statement

Where the words ‘women’, ‘maternal’ or ‘mother’ are used, these also refer to people who do not identify as women but have been pregnant or may be pregnant in the future.

### Scope

We defined multimorbidity in pregnancy as having two or more long-term physical or mental health conditions that pre-existed before pregnancy [1]. This core outcome set was developed to be applicable to research involving pregnant women with multimorbidity. It is not limited to specific long-term conditions, specific interventions or health care settings. The core outcome set would be applicable to observational and interventional studies.

### Study design

The core outcome set development protocol has been published [19] and registered in the Core Outcome Measures in Effectiveness Trials (COMET) database [20]. It follows the guidance of the COMET handbook [10] and involves four stages: (i) systematic literature search and (ii) focus groups to generate the initial list of outcomes; (iii) Delphi surveys and (iv) consensus meetings to prioritise the core outcomes. This report is prepared in accordance with the Core Outcome Set Standards for Reporting (COS-STAR, Additional file 1) [21].

### Participants

We recruited participants from the following stakeholder groups:

(i) Women with self-reported two or more long-term pre-existing conditions, who have been pregnant in the last 5 years or planning a pregnancy, and their partners
(ii) Health or social care professionals who provide care to pregnant women with multimorbidity or their children
(iii) Researchers interested in this field

Following the advice of our Patient and Public Involvement Advisory Group, we also recruited for partners, family and carers as they can provide a different perspective.

We contacted charities and organisations for health conditions, pregnancy, parenthood, health or social care professionals and researchers. We approached health condition-based charities guided by a list of 79 long-term conditions from our prior work [1]. We asked if they would share the study adverts with their members and through their social media platforms. We also recruited participants through professional contacts and networks.

### Systematic literature search

The systematic literature search was conducted in two stages. We first searched for published core outcome sets for multimorbidity, pregnancy and childbirth in the COMET and Core Outcomes in Women’s and Newborn’s Health (CROWN) databases. We then searched for studies of pregnant women with multimorbidity in Medline, Embase, Cumulated Index to Nursing and Allied Health Literature (CINAHL) and Cochrane Library from inception to 11 August 2021. We used the concepts ‘pregnancy’ (population) and ‘multimorbidity’ (exposure) to inform the search strategy using Medical Subject Headings and free text terms. Studies that reported outcomes for pregnant women with multimorbidity or their children were included. Two reviewers (SIL and MS) independently screened the full texts and extracted the types of outcomes reported in the studies. Details of the literature search strategy and study selection are provided in Additional file 2 [22–24]. As no evidence synthesis was undertaken, the quality of included studies was not assessed.

### Focus groups

As outcomes identified in the literature may be more representative of outcomes that are of interest to researchers rather than women or other stakeholders, we supplemented the initial list of outcomes with qualitative studies (focus groups) involving stakeholders [10]. The findings from the focus groups will be reported in more detail in a separate publication [25]. Briefly, three focus groups were conducted in the UK from December 2021 to March 2022: one for women, one for women and their partners and one for health professionals. Participants were recruited through study adverts disseminated through social media platforms of patient charities and professional organisations. We undertook maximum variation purposive sampling to ensure representation from different health conditions, ethnic groups, under-served populations, UK regions, availability of partners and specialties of health care professionals [19]. The focus groups explored outcomes that stakeholders felt should be reported in all studies of pregnant women with multimorbidity. Thematic analysis was conducted with an inductive approach [26], focusing on research outcomes discussed or inferred by participants. Outcomes from the focus groups were then compared to outcomes extracted from the systematic literature search to identify new outcomes.

### Delphi surveys

Prior to designing the Delphi surveys, two workshops were convened with the multidisciplinary research team and Patient and Public Advisory Involvement Group: one for maternal outcomes and one for child outcomes. The aim of the workshops was to review and refine the initial list of outcomes from the systematic literature search and focus groups. To reduce survey burden, we prioritised outcomes that clinicians and patient representatives felt are of higher risk in women with multimorbidity than women with no or single health condition. Outcomes that were clinically and pathophysiologically similar were combined. Important outcomes that were missing were added. The refined list of outcomes was then further prioritised by stakeholders through two rounds of Delphi surveys.

The Delphi surveys were piloted by the research team and Patient and Public Involvement Advisory Group and amended for clarity. A plain English explanation of medical terminology was provided in the survey, reflecting terminology used by participants in the focus groups where possible. For each outcome, participants were asked to rate on a 9-point scale (1–3 *Not important*; 4–6 *Important but not critical*, 7–9 *Critically important*). There was an *Unable to comment* option. Participants’ demographics were collected to iteratively inform the recruitment strategy.

The Delphi survey was in English and was hosted on https://www.onlinesurveys.ac.uk/. The study advert with a direct link to the survey was shared through patient charities and professional organisations’ social media network internationally. The targeted sample size was 50 women and 50 health or social care professionals based on previous studies [11, 12, 19, 27]. The first survey was opened from 28 April 2022 to 19 June 2022. Participants were invited to suggest up to two additional outcomes. New outcomes that were suggested by two or more participants were included in the second survey.

The second survey was opened from 24 June 2022 to 1 August 2022. Participants who took part in the first survey were sent personalised emails to take part in the second survey. All outcomes from the first survey were presented again. Participants were asked to reflect on the findings from the first survey before rescoring the outcomes [10]. They were given their individual scores and the aggregate scores across stakeholder groups (all participants, women/partners and health professionals/researchers). These were presented as median scores and percentages of participants rating the outcomes as *Critically important*. As predefined in the study protocol [19], *Consensus in* was considered when outcomes were rated as *Critically important* by ≥70% of all participants (combining all stakeholder groups). Participants were also asked to indicate their interest in joining the consensus meetings.

Attrition analysis was conducted to assess the impact of attrition from the second Delphi survey. For each outcome in the first Delphi survey, Mann-Whitney test was performed to compare the average scores [10], chi-squared test was performed to compare the proportion of participants who rated an outcome as *Critically important.* Comparisons were made between participants who completed the first survey only and participants who completed both rounds of the survey [10].

### Consensus meetings

For both meetings, we sampled participants from the second survey, focus groups, the research team and Patient and Public Involvement Advisory Group. Participants that were available were sampled with maximum variation to ensure representation from different stakeholder groups, specialty and geographical regions [19]. Similar to previous studies, and to facilitate discussion, we aimed to recruit 10 to 15 participants [11, 28, 29].

#### First consensus meeting

The first consensus meeting discussed outcomes that were considered *Borderline.* Outcomes were considered *Borderline* if in the second survey: (i) ≥70% of all participants rated the outcome as *Important but not critical,* or (ii) when ≥70% of participants in one stakeholder group (women/partner or health professionals/researchers) rated an outcome as *Critically important* but *Consensus in* was not reached. Participants were asked to review the list of *Borderline* outcomes before the meeting.

The virtual meeting took place in September 2022 and was facilitated by a non-voting chair (SIL, public health). It was conducted following the principles of a nominal group technique [10]. Participants voiced their opinions in turn without being interrupted in the *Round robin* session. This was followed by a *Group discussion* where participants could ask for clarifications from fellow participants. After hearing everyone’s views, the meeting ended with a final binary vote, *Prioritisation*. *Borderline* outcomes that were voted in by ≥70% of all participants were included.

#### Second consensus meeting

The second consensus meeting reviewed all the outcomes that were included from the second Delphi survey and the first consensus meeting. Pre-meetings were arranged with all participants to brief on the aim of achieving a concise core outcome set. Participants were asked to review the list of outcomes before the meeting.

The meeting was conducted virtually in February 2023; the non-voting co-chairs were MB (obstetrician) and CG (neonatologist). The group discussion focused on which outcomes had overlapping concepts and could be combined. Following the group discussion, a formal vote was held for maternal and child outcomes. The results were reviewed with further discussion, especially where there was no outcome included for key domains and where there was discrepancy of votes between stakeholder groups. This was followed by four additional rounds of voting. The criteria for inclusion were set before the meeting as ≥80% of yes votes from all participants.

## Results

### Changes to the protocol

Changes were made to the systematic literature search, number of rounds for the Delphi survey, the number and scope of the consensus meetings and the criteria for inclusion in the consensus meetings. The systematic literature search for studies reporting outcomes for pregnant women with multimorbidity from inception to 11 August 2021 identified 18,962 titles. Due to this large yield, study selection and data extraction were performed on a yearly basis until saturation was reached (when no new outcomes were extracted). We encountered difficulties recruiting women and partners for the first Delphi survey. We anticipated that the imbalance of stakeholders may widen with attrition in subsequent surveys. Therefore, we reduced the Delphi surveys from the planned three rounds to two rounds [19] and conducted a post hoc attrition analysis. Despite confirming attendance from equal numbers of stakeholders, there was an imbalance of stakeholder representation at the first consensus meeting. Following the advice of women stakeholders, we additionally included one outcome that was voted in by ≥70% of women stakeholders in the first consensus meeting. Finally, given the long list of included outcomes at the end of the first consensus meeting, a second consensus meeting was conducted to further prioritise outcomes, and the inclusion threshold was increased to ≥80% of all participants voting for the outcome.

### Initial list of outcomes

Additional file 3 presents the PRISMA flow chart for the systematic literature search, characteristics of included studies, reasons for exclusions, extracted outcomes and definitions [4, 5, 15, 16, 18, 30–69]. The search in COMET and CROWN identified one core outcome set for multimorbidity [18]; two for maternity care, pregnancy and childbirth [15, 16]; and five systematic reviews [56–60]. For studies reporting outcomes for pregnant women with multimorbidity, 7534 titles and abstracts from 2017 to 2021 were screened, 32 full texts were assessed for eligibility and three additional articles were included from screening the reference list of the included articles. A total of 28 articles were included from 26 studies [4, 5, 30–55].

**Fig. 1:**
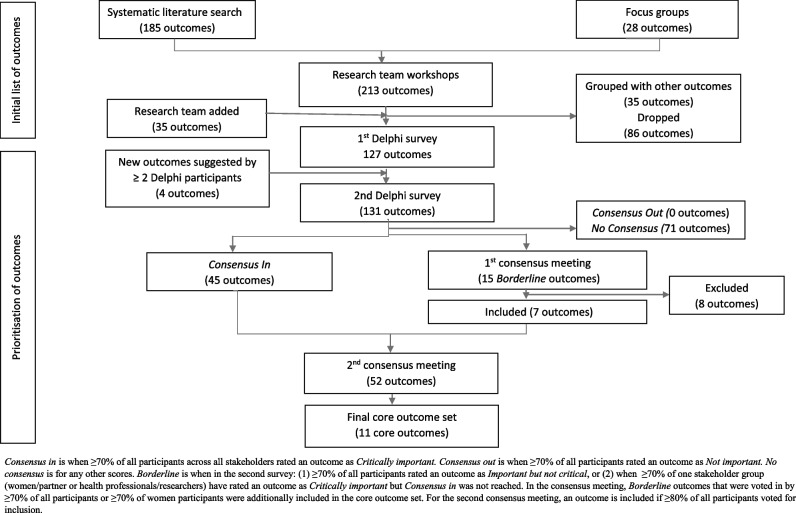
Flowchart of outcomes selection

From the systematic literature search, 185 unique outcomes were identified. The focus groups identified 63 outcomes; when mapped to the systematic literature search, 28 outcomes were new [25]. These 213 outcomes were reviewed in workshops with the research team and patient representatives; 35 outcomes, including seven outcomes from a core outcome set for neonatal research [28], were added; 86 outcomes were dropped and 35 outcomes were combined with other outcomes, giving a total of 127 outcomes for the first Delphi (Fig. 1). Additional file 4 lists the initial outcomes and rationale for decisions from the research team’s workshops.

### Delphi surveys

Table 1 shows the characteristics of the survey participants. In the first survey, 209 participants took part: 62 women, one partner, 102 health professionals and 44 researchers. In the second survey, 116 participants took part: 38 women, one partner, 52 health professionals and 25 researchers. In the first survey, 19 women/partner and 77 health professionals/researchers were from non-European countries; in the second survey, 12 women/partner and 34 health professionals/researchers were from non-European countries. The overall attrition rate was 44%: 39% for women, 49% for health professionals and 43% for researchers.

**Table 1:** Characteristics of participants

**Characteristics**	**1**^**st**^** Delphi, *n***	**2**^**nd**^** Delphi, *n***	**1**^**st**^** consensus meeting, *n***	**2**^**nd**^** consensus meeting, *n***
**Total**	209	116	13	17
**Stakeholders**				
Service users: Women with multiple long-term conditions	62	38	6	9
Service users: Partner	1	1	-	-
Service providers: Health or social care professionals	102	52	7	8
Researchers	44	25	(5 health professionals have dual roles as researchers)	(8 health professionals have dual roles as researchers)
**Consensus meeting recruitment**				
From focus group and Delphi surveys participants	**-**	**-**	8	11
From research team	**-**	**-**	3	1
From Patient and Public Involvement Advisory Group	**-**	**-**	2	5
**Geography**				
Africa	51	20	1	1
Asia	23	14	-	-
Australia and New Zealand	7	5	-	-
Europe	112	70	12	16
Middle East	3	1	-	-
North America	11	5	-	-
South America	1	1	-	-
Prefer not to say	1	-	-	-
**Urban/rural**				
Urban	169	97	11	15
Rural	35	15	2	1
Prefer not to say	5	4	-	1
**Ethnicity**				
Asian	40	26	-	1
Black, Caribbean or African	48	21	1	2
Mixed or multiple ethnic groups	3	1	-	-
White	110	66	11	13
Other	4	-	-	-
Prefer not to say	4	2	1	1
**Age in years**				
Median (interquartile range)	36 (31 to 44)	37 (32 to 47)	42 (32 to 44)	41 (34 to 46)
Range	22 to 70	23 to 70	28 to 70	28 to 61
Prefer not to say/missing	4	3	1	1
**Woman stakeholders**				
Pregnant in the last 5 years	54	33	3	3
Planning a pregnancy	8	5	1	1
Patient and Public Involvement Advisory Group	-	-	2	5
***Number of health conditions (median, IQR)***	3 (2 to 4)	3 (2 to 4)	4 (2 to 6)	3 (2 to 4)
***Number of health conditions (range)***	2 to 11	2 to 11	2 to 11	2 to 11
***Health conditions***				
Mental health conditions	29	17	4	4
Rheumatology/musculoskeletal	21	13	4	3
Gastroenterology	16	6	-	1
Endocrine	15	10	-	-
Respiratory	13	8	1	1
Neurology	12	8	2	3
Women’s health	12	7	-	1
Cardiovascular	12	6	1	-
Dermatology/allergies	11	5	-	1
Other	5	3	1	2
Neurodevelopmental	5	2	1	2
Haematology	4	4	1	1
Genetic	4	3	-	-
***Under-served characteristics*** (Includes addiction, asylum seeker, disabled, homeless/supported accommodation, LGBTQ+, migrant, victims of domestic abuse, other)	18	14	5	8
***Education***				
Primary	1	1	-	-
Secondary	10	7	-	1
Tertiary	46	27	6	7
Vocational	4	2	-	1
Other	-	-	-	-
Prefer not to say	1	1	-	-
**Health or social care professional stakeholders**				
Midwife/nurse/health visitor	39	19	4	3
Obstetrician/maternal and fetal medicine specialist	19	9	1	1
Obstetric physician/physician/anaesthetist	15	10	-	1
Family medicine/general practitioner	9	5	1	1
Paediatrician/neonatologist	7	4	1	1
Psychiatrist/perinatal mental health specialist/psychotherapist	5	2	**-**	1
Other	5	1	**-**	-
Not stated	3	2	**-**	-
**Researcher stakeholders’ area of research**				
Maternal and infant health/midwifery/obstetrics/women’s/reproductive health	24	14	5	4
Epidemiology/pharmacoepidemiology	6	-	-	-
Primary care/nursing	3	3	-	1
Medical specialties	2	2	-	2
Psychiatry/psychology	2	2	-	1
Not stated	7	4	-	-

*IQR* Interquartile range, *LGBTQ+* Lesbian, gay, bisexual, transgender, queer and others

In the first survey, 42 outcomes reached *Consensus in.* The list of additional outcomes suggested by participants is provided in Additional file 5. Four outcomes were suggested by two or more participants and were added to the second survey. These were as follows: cephalopelvic disproportion, childhood vaccination, feeding support and neonatal abstinence syndrome. In the second survey, 45 outcomes reached *Consensus in* (Table 2). In the attrition analysis, using a 5% significance level, three outcomes reached significance in the Mann-Whitney test and six outcomes reached significance in the chi-squared test. These different scoring patterns did not change whether the outcomes reached *Consensus in* in the first Delphi. Additional file 5 presents the percentage of participants that rated the outcomes as *Critically important*, stratified by stakeholder groups and the attrition analysis.

**Table 2:** Fifty-two preliminary outcomes included in the second Delphi survey and first consensus meeting

**Maternal outcomes**	**Children’s outcomes**
***Survival*** 1. Maternal death ***Clinical: antenatal*** 2. Miscarriage^a^ 3. Termination of pregnancy^a^ 4. Pre-eclampsia, eclampsia, HELLP syndrome 5. Placenta abruption 6. Placenta insufficiency 7. Venous thromboembolism ***Clinical: peripartum*** 8. Preterm premature rupture of membrane 9. Severe maternal morbidity 10. Postpartum haemorrhage 11. Hysterectomy 12. Maternal infection ***Clinical: postpartum and longer term*** 13. Development of new long-term conditions 14. Impact on long-term conditions ***Resource use/care-related outcomes*** 15. Admission to intensive care unit 16. Involvement in care decisions (overall care) 17. Involvement in care decisions (types of birth)^a^ 18. Postpartum admission/readmission 19. Quality and experience of care^a^ 20. Care for long-term conditions^a^ ***Mental health*** 21. Suicide (perinatal) 22. Post-traumatic stress disorder 23. Perinatal mental health 24. Self-harm (perinatal) 25. Perinatal mental health support	***Survival*** 1. Death before birth (intrauterine death, stillbirth, perinatal death) 2. Death after birth (neonatal death, infant death) ***Clinical: fetal*** 3. Fetal growth restriction ***Clinical: neonatal*** 4. Gestational age at birth 5. Apgar score 6. Birth weight 7. Neonatal resuscitation required 8. Requiring intubation/ventilation 9. Neonatal birth injury 10. Neonatal sepsis 11. Brain injury on imaging 12. Neonatal respiratory distress syndrome 13. Necrotizing enterocolitis 14. Retinopathy of prematurity 15. Neonatal abstinence syndrome 16. Meconium aspiration syndrome 17. Separation of mother from baby^a^ ***Clinical: infant*** 18. Chronic lung disease/bronchopulmonary dysplasia ***Clinical: longer term*** 19. Congenital anomaly 20. Cerebral palsy 21. Children mental health and behavioural disorder 22. Need for complex care 23. Neurodevelopmental conditions ***Life impact/functioning*** 24. Visual impairment/blindness 25. Quality of life^a^ ***Resource use*** 26. Admission to neonatal unit (including intensive care) 27. Neonatal readmission to hospital

*HELLP* Haemolysis, elevated liver enzymes and low platelets

^a^The seven *Borderline* outcomes that were included after discussion in the first consensus meeting

### First consensus meeting

From the second survey, 15 *Borderline* outcomes were eligible for discussion at the first consensus meeting. Thirteen participants took part in the meeting: six women and seven health professionals/researchers (Table 1). Additional file 6 presents the meeting minutes and the votes for these 15 *Borderline* outcomes; seven additional outcomes were included (Table 2).

### Second consensus meeting

The 52 outcomes included from the second Delphi survey and first consensus meeting were discussed (Table 2). Seventeen participants took part: nine women and eight health professionals/researchers (Table 1). Additional file 7 presents the meeting minutes and the voting results. The final core outcome set included 11 outcomes: five maternal and six child outcomes. Table 3 presents the final list of core outcomes and key points from the discussion. These should be considered in the next stage when determining how the core outcomes should be defined and measured.

**Table 3:** Eleven core outcomes in the final core outcome set for studies of pregnant women with multimorbidity

**Core outcomes**	**Concepts of the outcomes and key points for consideration in the next stage of defining outcomes**
**Maternal outcomes**	
1. Maternal death	Important to document timing and cause of death.
2. Severe maternal morbidity	Many of the pregnancy complications that were initially included were removed from the core outcome set as severe maternal morbidity would represent the severe manifestation of the pregnancy complications.
3. Change in existing long-term conditions (physical and mental)	Includes the worsening, relapse or improvement of pre-existing long-term physical and mental health conditions.
4. Quality and experience of care	Important to include whether women were involved in their care decisions.
5. Development of new mental health conditions	This would include the development of new onset mild, moderate and severe mental health conditions that are acute or chronic.
**Child outcomes**	
1. Survival of baby	To include early pregnancy loss (miscarriage) and death of the baby at different time points (e.g. intrauterine fetal demise, stillbirth, perinatal death, neonatal death, infant death). Important to include the time frame, e.g. death within 28 days for neonatal death.
2. Gestational age at birth	This outcome together with birth weight and sex can be used to derive other outcomes, such as preterm/post-term birth, small/large for gestational age, fetal growth restriction.
3. Neurodevelopmental conditions/impairment	Important to determine what is the definition, what conditions to include, and the severity level at which it impairs function. Important to ensure research is conducted ethically.
4. Quality of life	Will need the development of measurement tools to measure this outcome in very young babies.
5. Birth weight	Studies should also document the sex of the baby alongside this outcome to enable meaningful interpretation.
6. Separation of baby from mother for health care needs	This would be a proxy for baby or mother needing additional care, such as admission to neonatal unit or intensive care unit.

#### Consensus meetings key discussion points

In the consensus meetings, participants spoke about the importance of exploring the reasons behind women having a *Termination of pregnancy*, whether women received good support and counselling for this decision, and whether women felt coerced.

*Neurodevelopmental conditions (child)* reached *Consensus in* in the second survey, whilst *General cognitive ability (child)* was considered *Borderline* and was ultimately not voted in the first consensus meeting. The opinions for these two outcomes were split in the consensus meetings. Participants who did not support the inclusion of these outcomes were concerned that it will lead to study findings that encourage ableism, place the blame of these child outcomes on women’s choices and limit women’s access to certain medication or types of birth. Participants who supported the inclusion of these outcomes felt that having information on these outcomes is important for pregnant women with multimorbidity to make informed decisions for their care during pregnancy. This includes decisions on medications they take during pregnancy and their babies’ treatment during the neonatal period.

There was general agreement that the perinatal mental health outcomes needed to be combined and to be included in the core outcome set. However, there were debates on whether the core outcome set should focus on mental health conditions that are severe. Participants raised concerns that, depending on the definition of severe mental health conditions used, this may not capture birth-related post-traumatic stress disorder.

*Separation of baby from mother* overlapped with *Admission to neonatal unit*. Women participants felt very strongly for the former. They were concerned about the long-term impact on the baby if admission to neonatal unit was required, but additionally spoke about the anxiety that came with the separation. *Separation of baby from mother* may also overcome the challenges of international variation in how neonatal care is provided.

## Discussion

### Main findings

This paper reports the process of developing a core outcome set for studies of pregnant women with multimorbidity. The final core outcome set included 11 outcomes: five maternal outcomes and six child outcomes. Maternal outcomes covered survival, severe manifestation of maternal complications during pregnancy and childbirth, impact on the women’s multiple long-term conditions and mental health and experiential outcomes. Child outcomes covered survival, gestational age and birth weight, separation of baby and mother at birth for health care needs and longer-term neurodevelopmental and quality of life outcomes.

### Comparison with the literature

Outcomes that are of importance to all pregnant women are likely to also be important to pregnant women with multimorbidity. Therefore we expected an overlap of the current core outcome set with existing core outcome set for pregnancy, childbirth and maternity care [15, 16], such as survival of mother and child, gestational age at birth, birth weight and quality and experience of care. *Severe maternal morbidity* that arises during childbirth, a composite outcome that is frequently used in recent USA-based studies of maternal multimorbidity, was also included [3, 4, 40, 42, 44]. However, our study additionally included core outcomes specific to pregnant women with multimorbidity such as *Change in existing long-term conditions (physical and mental).*

### Strengths and limitations

The core outcome set was developed with a robust multistage approach, balancing the views of all stakeholders including women with multimorbidity, health and social care professionals and researchers. The broad remit of multimorbidity allowed us to work with many national and international patient charities for recruitment. This is reflected in the broad range of study participants, including participants from under-served groups, who provided invaluable perspective on the included outcomes. The multidisciplinary nature of maternal multimorbidity was also reflected in the range of health professionals who participated, including health professionals in women’s health, children’s health and mental health, in both primary care and hospital settings.

Our Patient and Public Involvement Advisory Group was involved at all stages of the study. This is a diverse group of women with lived experience of a broad range of health conditions, disabilities, geographical and ethnic representation in the UK. They advised on the scope of the core outcome set, reviewing and piloting the study materials, recruitment, conduct of the study, interpreting the focus group findings, selection of the initial outcomes and participating in the consensus meetings.

However, a key limitation of this study is the representation of stakeholders. Despite having more women stakeholders in the focus groups, only a third of the Delphi surveys participants were women stakeholders. Although a third of women/partner stakeholders who participated in the Delphi surveys were from non-European countries, all women stakeholders at the consensus meeting were based in the UK. The study findings may not represent the views of participants who do not have digital access or experience care outside of the UK or similar high-income settings.

Despite recruiting for family members, carers and partners of women with multimorbidity, only two partners participated in the focus groups and one partner participated in the Delphi surveys. We were not able to consider the views of children born to mothers with multimorbidity. It may be possible that some of the women participants met these criteria given the hereditary nature of some health conditions, but this information was not captured. The WRISK study highlighted concerns that current pregnancy risk messaging prioritises fetal health over the women’s health outcomes [70, 71]. Therefore, this study focuses on maternal and child outcomes that are important to women with multimorbidity and information that will help women make informed decisions for their own care during pregnancy and in the postpartum period.

The attrition rate in the follow-up survey was high (44%). Previous studies have reported attrition rates ranging from 21 to 48% [29, 60]. The survey burden presented by the long list of outcomes is likely to have contributed to the difficulty in recruitment and retention. To avoid further imbalance of stakeholder representation, we terminated the Delphi survey after the second round. This meant participants did not have the opportunity to reflect on the scores for the four new outcomes added from the first Delphi survey.

### Research implications

A core outcome set lists the minimum standard list of outcomes that should be measured (‘what to measure’). Once this is defined through consensus-setting methods, a separate piece of work is needed to reach consensus on how the core outcomes should be defined and measured (‘how to measure’) [10], following the guidance of the Consensus-based Standards for the Selection of Health Measurement Instruments (COSMIN) initiative [72]. This includes a literature search to identify existing measurement instruments for each of the core outcomes, quality assessment of the instruments and a consensus process to agree on one instrument per core outcome. In this study, key points were raised in the consensus meetings on defining the core outcomes, these should be taken into consideration when developing a consensus on how to measure the 11 core outcomes. The next step is to disseminate the core outcome set for use in future observational and interventional studies in line with the Standard Protocol Items: Recommendations for Interventional Trials (SPIRIT) statement and the CROWN initiative [9, 73]. As this core outcome set is also applicable to observational studies using routine health records, it can be considered by those designing data collection tools within the healthcare services. This can provide consistency in data collection across healthcare providers, allowing for clinical audit and secondary analysis.

To reduce the survey burden, some outcomes were combined into broader categories when designing the Delphi surveys. For instance, vaginal, caesarean and instrumental births were combined as *Types of birth*. Preterm births, small and large for gestational age, are captured by *Gestational age* and *Birth weight.* Some outcomes were considered so important they were kept as standalone outcomes alongside broader outcomes, such as *Cerebral palsy*, *General motor*, *cognitive* and *social ability* alongside *Neurodevelopmental conditions (child)*; *Post-traumatic stress disorder*, *Suicide*, *Self-harm* alongside *Perinatal mental health*. Although we have grouped all types of *Congenital anomaly* and *Neurodevelopmental conditions* into one outcome respectively, that does not mean they should be researched as one entity. Depending on the research question and granularity of the data source, further subclassification of the types of congenital anomaly and neurodevelopmental conditions may be required.

Some of the outcomes were process measures. The second consensus meeting offered the opportunity to consider whether these process measures or the associated longer-term impact are more important. For instance, the quality of *Care for long-term conditions* and *Perinatal mental health support* would ultimately determine the status of the women’s long-term conditions or mental health outcomes; *Requiring intubation/ventilation (neonate)* and *Neonatal resuscitation* matter if the baby required admission to the neonatal unit is separated from the mother or develops longer-term complications. Consequently, many of these process measures were not included in the final core outcome set.

In the consensus meeting, some women stakeholders were concerned about the introduction of ableism through child outcomes such as *Neurodevelopmental conditions.* Ableism is a value system that discriminates against people with disabilities [74]. Disabled people have differing views, some may find research aimed at preventing impairment offensive whilst others are supportive [74]. The term neurodevelopmental conditions itself has been widely debated. Within the spectrum of neurocognitive function, there are neurodivergent individuals whose neurocognitive differences fall outside societal norms but are not considered impairment, whilst a diagnosis of neurodevelopmental conditions is for those with significant functional impairment [75]. It is, therefore, imperative to keep an open conversation with disabled people and maintain sensitivity and awareness about this [76]. It is also important to involve people with neurodevelopmental conditions in research about the condition itself [77].

The inclusion of perinatal mental health outcomes is important as it is one of the commonest complications of pregnancy, with suicide being the leading cause of maternal death, especially in high-income countries [78–80]. Severe mental health conditions were proposed as an umbrella outcome for perinatal mental health outcome and were discussed at length. Health professionals wanted to focus on mental health conditions that are severe. However, women participants were concerned that this would not capture birth-related post-traumatic stress disorder. There is no international consensus on the definition of severe mental illness/health conditions [81]. Conventionally, two approaches are being used: narrow (three-dimensional) and broad (two-dimensional) operationalised definitions of severe mental health conditions [81, 82]. The three dimensions consider the following: (i) a diagnosis of non-organic psychosis, (ii) duration and (iii) disability [81, 82]. The first approach includes a narrower list of health conditions (e.g. bipolar affective disorder, schizophrenia, psychosis) and is widely used in health services and research [83, 84]. The second approach uses the latter two dimensions and would include any mental health conditions resulting in serious functional impairment [85]; it was advocated by health professional participants.

As discussed by Zumstein et al., although international consensus for severe mental health conditions can facilitate large-scale epidemiological studies, definitions that are context-specific may be more useful [82]. For example, in the context of perinatal mental health, health professional participants raised the difficulty with the duration criteria, which may exclude acute perinatal mental health conditions which are nevertheless severe. Ultimately, two of the included core outcomes will capture perinatal mental health outcomes: *Change in existing long-term conditions* will capture improvement, worsening, or relapse of existing mental health conditions; *Development of new mental health conditions* will capture new onset antenatal and postnatal mental health conditions, such as birth-related post-traumatic stress disorder, self-harm and suicide attempts, postnatal depression and puerperal psychosis.

Finally, just because an outcome is not included in the core outcome set does not mean it is not important. Additional study-specific outcomes can still be measured depending on the research question. This can be guided by the preliminary list of 52 outcomes prioritised by stakeholders through the Delphi surveys and first consensus meeting. For instance, studies of medication safety in pregnant women with multimorbidity may want to include *Congenital anomaly (child)* [17]. As more studies are conducted for pregnant women with multimorbidity, an update of this core outcome set may be indicated in the future [10].

## Conclusions

Multimorbidity in pregnancy is a new and complex clinical research area. Developing a core outcome set for studies of pregnant women with multimorbidity requires broader inclusion of participants. Following a rigorous process, this complexity was meaningfully reduced to a core outcome set that balances the views of a diverse stakeholder group. It included outcomes for obstetrics, maternity services, perinatal mental health, maternal long-term conditions and child outcomes, reflecting the multidisciplinary nature of multiple long-term conditions in pregnancy.

### Supplementary Information

**Additional file 1.** COS-STAR checklist.

**Additional file 2.** Methods for the systematic literature search.

**Additional file 3.** The PRISMA flow chart for the systematic literature search, characteristics of included studies, studies that were excluded and reasons for exclusion (stage 2), and summary list of extracted outcomes.

**Additional file 4.** Initial outcomes and rationale for decisions from the research team’s workshops.

**Additional file 5.** Results from the Delphi surveys and attrition analysis.

**Additional file 6.** First consensus meeting report.

**Additional file 7.** Second consensus meeting report.

## Data Availability

All data generated or analysed during this study are included in this published article and its additional files.

## Abbreviations

CINAHL: Cumulated Index to Nursing and Allied Health Literature
COMET: Core Outcome Measures for Effectiveness Trials
COSMIN: Consensus-based Standards for the Selection of Health Measurement Instruments
COS-STAR: Core Outcome Set Standards for Reporting
CROWN: Core Outcomes in Women’s and Newborn Health
HELLP: Haemolysis, elevated liver enzymes and low platelets
IQR: Interquartile range
LGBTQ+: Lesbian, gay, bisexual, transgender, queer and others
PRISMA: Preferred Reporting Items for Systematic Reviews and Meta-Analyses
SPIRIT: Standard Protocol Items: Recommendations for Interventional Trials
UK: United Kingdom
