# Supplementary material for: The development of a core outcome set for studies of pregnant women with multimorbidity
Source: BMC Med. 2023 Aug 21;21:314. doi: 10.1186/s12916-023-03013-3 (PMC10441728; doi:10.1186/s12916-023-03013-3)
Supplement: Supplementary file 1 — Additional file 1. COS-STAR checklist. [file 12916_2023_3013_MOESM1_ESM.docx]

## Additional File 1: COS-STAR checklist

| **Section / Topic** | **Item No.** | **Checklist Item** | **Manuscript** |
| --- | --- | --- | --- |
| **TITLE / ABSTRACT** |  |  |  |
| Title | 1a | Identify in the title that the paper reports the development of a COS | Page 1: Title: Core outcomes for studies of pregnant women with multiple long-term conditions (multimorbidity) and their children: development of a core outcome set |
| Abstract | 1b | Provide a structured summary | Page 3 |
| **INTRODUCTION** |  |  |  |
| Background and  Objectives | 2a | Describe the background and explain the rationale for developing the COS. | Page 5: Background |
|  | 2b | Describe the specific objectives with reference to developing a COS. | Page 6: Last sentence of the Background sentence states the aim of the study. |
| Scope | 3a | Describe the health condition(s) and population(s) covered by the COS. | Page 6: Scope |
|  | 3b | Describe the intervention(s) covered by the COS. | Page 6: Scope |
|  | 3c | Describe the setting(s) in which the COS is to be applied. | Page 6: Scope |
| **METHODS** |  |  |  |
| Protocol/Registry Entry | 4 | Indicate where the COS development protocol can be accessed, if  available, and/or the study registration details. | Page 7: Study design |
| Participants | 5 | Describe the rationale for stakeholder groups involved in the COS  development process, eligibility criteria for participants from each  group, and a description of how the individuals involved were identified. | Page 7: Participants |
| Information Sources | 6a | Describe the information sources used to identify an initial list of  outcomes. | Page 8: Systematic literature search  Page 8: Focus groups |
|  | 6b | Describe how outcomes were dropped/combined, with reasons (if  applicable). | Page 9: Delphi surveys  Supplementary material 4: Selection of initial list of outcomes for Delphi surveys (workshop) |
| Consensus Process | 7 | Describe how the consensus process was undertaken. | Page 9-12: Delphi surveys, Consensus meetings |
| Outcome Scoring | 8 | Describe how outcomes were scored and how scores were  summarised. | Page 9-12: Delphi surveys, Consensus meetings |
| Consensus Definition | 9a | Describe the consensus definition. | Page 9-12: Delphi surveys, Consensus meetings |
|  | 9b | Describe the procedure for determining how outcomes were included or  excluded from consideration during the consensus process. | Page 9-12: Delphi surveys, Consensus meetings  Figure 1 |
| Ethics and Consent | 10 | Provide a statement regarding the ethics and consent issues for the study. | Page 30: Declarations: Ethics and consent |
| **RESULTS** |  |  |  |
| Protocol Deviations | 11 | Describe any changes from the protocol (if applicable), with reasons,  and describe what impact these changes have on the results. | Page 12: Changes to the protocol |
| Participants | 12 | Present data on the number and relevant characteristics of the people  involved at all stages of COS development. | Table 1: Characteristics of participants |
| Outcomes | 13a | List all outcomes considered at the start of the consensus process. | Supplementary material 5: Delphi results |
|  | 13b | Describe any new outcomes introduced and any outcomes dropped,  with reasons, during the consensus process. | Page 14: Delphi surveys  Supplementary material 5: Delphi results |
| COS | 14 | List the outcomes in the final COS. | Table 3 |
| **DISCUSSION** |  |  |  |
| Limitations | 15 | Discuss any limitations in the COS development process. | Page 17-18: Strengths and limitations |
| Conclusions | 16 | Provide an interpretation of the final COS in the context of other  evidence, and implications for future research. | Page 19-21: Research implications |
| **OTHER**  **INFORMATION** |  |  |  |
| Funding | 17 | Describe sources of funding/role of funders. | Page 30: Declarations: Funding |
| Conflicts of Interest | 18 | Describe any conflicts of interest | Page 30: Declarations: Competing interest |
