# Supplementary material for: The development of a core outcome set for studies of pregnant women with multimorbidity
Source: BMC Med. 2023 Aug 21;21:314. doi: 10.1186/s12916-023-03013-3 (PMC10441728; doi:10.1186/s12916-023-03013-3)
Supplement: Supplementary file 2 — Additional file 2. Methods for the systematic literature search. [file 12916_2023_3013_MOESM2_ESM.docx]

## Additional File 2: Methods for the systematic literature search

**Search strategy**

In the protocol, we proposed to use the concepts of ‘pregnancy’ (population, outcome), ‘multimorbidity’ (exposure) and ‘offspring’ (outcome) for the search terms. However, in the scoping search, the search term ‘offspring’ resulted in studies that were conducted in children or adolescents with multimorbidity. The search strategy was modified to include the concepts for the targeted population (pregnancy) and exposure (multimorbidity), with the rationale that any outcomes for the targeted population would be captured in this broader search strategy. The search strategy was built with reference to previous literature [22, 23], using free-text terms and Medical Subject Headings (MeSH) terms (and its equivalent) for each database. No language limitation was applied. The reference lists of included studies were screened for additional studies.

| **Database** | **Search details** | **Search terms** |
| --- | --- | --- |
| **Stage 1: Published core outcome sets for multimorbidity and for pregnancy and childbirth in core outcome set databases** | | |
| COMET | Conducted on 15^th^ September 2021 | Disease name: Multimorbidity  Disease category: Pregnancy & childbirth |
| CROWN | Conducted on 7^th^ October 2021 | Hand searched |
| **Stage 2: Studies reporting outcomes for pregnant women with multimorbidity or their children** | | |
| Medline | Conducted on 11^th^ August 2021  Ovid Medline ( R ) and In-Process, In-Data Review & Other Non-indexed Citations, 1946 to 10^th^ August 2021 | **Multimorbidity**  1. (multimorbidity* or multi-morbidit* or comorbidit* or co-morbidit* or polymorbidit* or poly-morbidit* or multicondition* or multi-condition* or 'multiple chronic condition*' or 'morbidity burden' or ((multiple or coexisting or co-existing or concurrent or con-current or comorbid or co-morbid) adj2 (disease* or illness* or condition* or diagnos* or morbid*))).ti,ab. or exp Multimorbidity/ or exp Comorbidity/ (n=298,023)  **Pregnancy**  2. exp Pregnancy/ or exp Pregnant women/ or exp Gravidity/ or exp Mothers/ or exp Obstetrics/ or exp Delivery, obstetric/ or exp Parturition/ or exp Maternal Health/ or exp Maternal health services/ or (pregnan* or gravid* or gestation* or 'pregnant wom#n' or matern* or mother* or obstetric* or (child adj3 bearing) or childbearing or parturition or childbirth or child-birth or child birth).ti,ab. (n=1,343,221)  3. 1 and 2 (n=9297)  4. Limit 3 to human (n=8130) |
| Embase | Conducted on 11^th^ Aug 2021  Embase (Ovid) 1974 to 10^th^ Aug 2021 | **Multimorbidity**  1. (multimorbidity* or multi-morbidit* or comorbidit* or co-morbidit* or polymorbidit* or poly-morbidit* or multicondition* or multi-condition* or 'multiple chronic condition*' or 'morbidity burden' or ((multiple or coexisting or co-existing or concurrent or con-current or comorbid or co-morbid) adj2 (disease* or illness* or condition* or diagnos* or morbid*))).ti,ab. or exp Multiple Chronic Conditions/ or exp Comorbidity/ (n=525,241)  **Pregnancy**  2. exp Pregnancy/ or exp Pregnant Woman/ or Mother/ or exp Obstetrics/ or exp Obstetric Delivery/ or Birth/ or exp Childbirth/ or Maternal Care/ or (pregnan* or gravid* or gestation* or 'pregnant wom#n' or matern* or mother* or obstetric* or (child adj3 bearing) or childbearing or parturition or childbirth or child-birth).ti, ab. (n=1,455,813)  3. 1 and 2 (n=16,553)  4. Limit 3 to human (n=15,641) |
| CINAHL | Conducted on 11^th^ August 2021 | ID Search Hits  S1 (MH "Pregnancy+") 228,070  S2 (MH "Mothers+") 46,291  S3 (MH "Obstetrics") 6,412  S4 (MH "Delivery, Obstetric+") 15,558  S5 (MH "Maternal Health Services+") 33,349  S6 pregnan* OR gravid* OR gestation* OR “pregnant wom?n” 403,834  OR matern* OR mother* OR obstetric* OR (child N3 bearing)  OR childbearing OR parturition OR childbirth OR child-birth  OR “child birth”  S7 S1 OR S2 OR S3 OR S4 OR S5 OR S6 407,310  S8 (MH "Comorbidity") OR "multimorbidit* or multi-morbidit* 65,877  or comorbidit* or co-morbidit* or polymorbidit* or  poly-morbidit* or multicondition* or multi-condition* or  “multiple chronic condition*” or “morbidity burden” or  ((multiple or coexisting or co-existing or concurrent or  con-current or comorbid or co-morbid) N2 (disease* or  illness* or condition* or diagnos* or morbid*))"  S9 S7 AND S8 1,945 |
| Cochrane Library | Conducted on 11^th^ August 2021 | ID Search Hits  #1 MeSH descriptor: [Multimorbidity] explode all trees 56  #2 MeSH descriptor: [Comorbidity] explode all trees 3665  #3 (multimorbidity* OR multi-morbidit* OR comorbidit* OR co-morbidit* OR polymorbidit* OR poly-morbidit* OR multicondition* OR multi-condition* OR “multiple chronic conditions” OR “morbidity burden” OR ((multiple OR coexisting OR co-existing OR concurrent OR con-current OR comorbid OR co-morbid) NEAR/2 (disease* OR illness* OR condition* OR diagnos* OR morbid*))):ti,ab 22058  #4 #1 OR #2 OR #3 24721  #5 MeSH descriptor: [Pregnancy] explode all trees 23021  #6 MeSH descriptor: [Pregnant Women] explode all trees 357  #7 MeSH descriptor: [Gravidity] explode all trees 62  #8 MeSH descriptor: [Mothers] explode all trees 1936  #9 MeSH descriptor: [Obstetrics] explode all trees 198  #10 MeSH descriptor: [Delivery, Obstetric] explode all trees 5420  #11 MeSH descriptor: [Parturition] explode all trees 468  #12 MeSH descriptor: [Maternal Health] explode all trees 69  #13 MeSH descriptor: [Maternal Health Services] explode all trees 2378  #14 (pregnan* OR gravid* OR gestation* OR (pregnant NEXT wom?n) OR matern* OR mother* OR obstetric* OR (child NEAR/3 bearing) OR childbearing OR parturition OR childbirth OR child-birth OR (child NEXT birth)):ti,ab 91692  #15 #5 OR #6 OR #7 OR #8 OR #9 OR #10 OR #11 OR #12 OR #13 OR #14 96928  #16 #4 AND #15 1088 |

CINAHL: Cumulative Index to Nursing and Allied Health Literature; COMET: Core Outcome Measures in Effectiveness Trials; CROWN: Core Outcomes in Women’s and Newborn Health

**Study selection criteria**

The following is the study selection criteria for stage 2 of the systematic literature search (studies reporting outcomes for pregnant women multimorbidity or their children). Title and abstract screening was conducted using Rayyan [24]. Full text screening was conducted by two researchers independently, using EndNote.

| No | Concept | Question |
| --- | --- | --- |
| 1 | **Population** | **Include**  Study identified or recruited the following population to study their outcomes:  - pregnant women with multimorbidity, OR  - children born to mothers with multimorbidity, OR  - general population of pregnant women and compared the outcomes of those with multimorbidity versus those with no multimorbidity  **Exclude**  - Participants who were identified or recruited based on the presence of an index condition (co-morbidity studies)  - Participants who were identified or recruited based on specific combination of diseases, that would limit their representation of pregnant women with multimorbidity  - Participants who were identified or recruited based on the presence of an outcome  **Definition of multimorbidity**  - 2 or more long-term physical or mental health conditions  - Pre-existing long term conditions at conception, prior to pregnancy  - Does not include pregnancy related conditions or complications related to pregnancy such as gestational diabetes, pre-eclampsia  - *Severe maternal morbidity* refers to pregnancy complications and not pre-existing long term conditions; it is the outcome of interest not exposure of interest |
| 2 | **Exposure / intervention** | **Exposure:** Maternal multimorbidity that pre-existed prior to pregnancy  **Intervention**: Any intervention with the target population being pregnant women with multimorbidity  **Exclude**  - ‘Comorbidities’ adjusted as a covariate, confounder, effect modifier, mediator  - ‘Comorbidities’ refer to one disease or listed as individual diseases and not analysed as a combination of diseases or when it is not clear whether ‘comorbidities’ refers to multimorbidity |
| 3 | **Outcome** | **Include**  - any types of pregnancy / maternal / offspring outcomes (i.e., not limited to health outcomes)  NB. risk factors/ predictors/ factors associated with multimorbidity in pregnant women are not outcomes  **Exclude**  - Studies that have not collected / reported any outcomes  **Example outcomes based on the taxonomy of outcomes:**  i. *death –* mortality, survival  ii. *clinical/physiological –* e.g., cardiac outcome, psychiatric outcome  iii. *life impact –* physical/social/role/emotional/cognitive functioning, quality of life, adherence, satisfaction  iv. *resource use –* economic / hospital/ further intervention/carer burden  v. *adverse events* |
| 4 | **Study design / publication types** | **Include:**  - systematic reviews  - interventional studies / trials (randomised / non randomised controlled studies / quasi-experimental)  - observational (cohort / cross sectional)  - qualitative studies  - patient reported outcome measures (PROM) studies  - model validation study (if outcomes that the model of multimorbidity is trying to predict is reported)  **Exclude:**  - ongoing studies with no reported outcomes  - editorials / commentaries  - narrative reviews  - guidelines  - case reports / case series  - diagnostic accuracy studies – except tools to assess multimorbidity risk / status  - laboratory studies  - animal studies  NB.  - no language limitation  - abstracts / conference proceedings are also included if able to extract the types of outcomes the authors have collected / measured |
