# Supplementary material for: The development of a core outcome set for studies of pregnant women with multimorbidity
Source: BMC Med. 2023 Aug 21;21:314. doi: 10.1186/s12916-023-03013-3 (PMC10441728; doi:10.1186/s12916-023-03013-3)
Supplement: Supplementary file 3 — Additional file 3. The PRISMA flow chart for the systematic literature search, characteristics of included studies, studies that were excluded and reasons for exclusion (stage 2), and summary list of extracted outcomes. [file 12916_2023_3013_MOESM3_ESM.docx]

Table of Contents

[Additional File 3a: PRISMA flow chart of the systematic literature search 2](#_Toc141094823)

[Additional File 3b: Characteristics of the included studies 4](#_Toc141094824)

[Additional File 3c: Studies that were excluded and reasons for exclusion (stage 2) 12](#_Toc141094825)

[Additional File 3d: Summary list of extracted outcomes 13](#_Toc141094826)

## Additional File 3a: PRISMA flow chart of the systematic literature search

**Stage 1: COMET and CROWN database search results**

Search results

COMET database

- Multimorbidity (n=4)
- Pregnancy & Childbirth (n=112)

CROWN database (n=43)

Included

Published Core Outcome Set (COS, n=3)

Systematic review / literature review for COS (n=5)

COMET: Core Outcome Measures in Effectiveness Trials; CROWN: Core Outcomes in Women’s and Newborn Health

**Stage 2: Search results for studies reporting outcomes for pregnant women with multimorbidity or their children**

Articles removed *before screening*:

Duplicate records removed (n =7842)

Articles identified from:

Medline (n=8130)

Embase (n=15,641)

CINAHL (n=1945)

Cochrane (n=1088)

Total (n=26,804)

**Identification**

Articles excluded (n=7502)

2021 (n=1184) 2018 (n=1437)

2020 (n=1892) 2017 (n=1344)

2019 (n=1645)

After deduplication (n= 18,962)

Articles screened (n=7534)

2021 (n=1188) 2018 (n=1442)

2020 (n=1906) 2017 (n=1348)

2019 (n=1650)

Articles not retrieved

(n=0)

**Screening**

Articles sought for retrieval

(n=32)

Articles excluded (n=7):

Population: not pregnant women with multimorbidity (n=3)

Exposure: not multimorbidity (n=3)

Outcomes: no outcomes reported (n=1)

Articles assessed for eligibility

(n=32)

2021 (n=4) 2018 (n=5)

2020 (n=14) 2017 (n=4)

2019 (n=5)

Articles of included studies

(n=25)

2021 (n=2) 2018 (n=4)

2020 (n=11) 2017 (n=3)

2019 (n=5)

Additional eligible articles from the reference list of included articles (n=3)

**Included**

4 articles contributed to 2 studies, articles for same studies were combined (n=2)

Studies included for data extraction

(n=26)

Articles included for data extraction

(n=28)

## Additional File 3b: Characteristics of the included studies

**Stage 1: Search of the COMET and CROWN database for published core outcome sets**

| **Existing, published core outcome sets for multimorbidity** | |  |
| --- | --- | --- |
| **No** | **Reference** | **Comments** |
| 1 | Smith SM, Wallace E, Salisbury C, Sasseville M, Bayliss E, Fortin M. A Core Outcome Set for Multimorbidity Research (COSmm). Annals of family medicine. 2018;16(2):132-8. |  |
| **Existing, published core outcome sets for pregnancy** | |  |
| 2 | Devane D, Begley CM, Clarke M, Horey D, C OB. Evaluating maternity care: a core set of outcome measures. Birth (Berkeley, Calif). 2007;34(2):164-72 |  |
| 3 | Nijagal MA, Wissig S, Stowell C, Olson E, Amer-Wahlin I, Bonsel G, et al. Standardized outcome measures for pregnancy and childbirth, an ICHOM proposal. BMC Health Services Research. 2018;18(1):953. |  |
| **Systematic reviews / literature reviews for core outcome sets** | |  |
| 1 | Herman D, Lor KY, Qadree A, Horn D, D'Souza R. Composite adverse outcomes in obstetric studies: a systematic review. BMC pregnancy and childbirth. 2021;21(1):107. |  |
| 2 | Slavin V, Creedy DK, Gamble J. Core Outcome Sets Relevant to Maternity Service Users: A Scoping Review. Journal of Midwifery & Women's Health. 2021;66(2):185-202. | The review included 26 COS but only 1 COS was for pregnancy in general (the others were condition specific), which is already included (Devane 2007). |
| 3 | SBU Policy Support. Core outcome sets for research within the area of maternity care. Overview of completed and ongoing studies2020. Available from: <https://www.sbu.se/en/publications/sbu-bereder/core-outcome-sets-for-research-within-the-area-of-maternity-care/> | The review included 19 studies, 3 were for pregnancy in general, 2 were already included (Devane 2007, Nijagal 2018), 1 additional study prioritised maternity care quality indicators using the COS methodology was identified:  Bunch KJ, Allin B, Jolly M, Hardie T, Knight M. Developing a set of consensus indicators to support maternity service quality improvement: using Core Outcome Set methodology including a Delphi process. BJOG. 2018;125(12):1612-8. |
| 4 | Duffy J, Rolph R, Gale C, Hirsch M, Khan KS, Ziebland S, et al. Core outcome sets in women's and newborn health: a systematic review. BJOG. 2017;124(10):1481-9. | The review identified 20 systematic reviews and 4 COS published, of which 1 systematic review (Smith 2014) and 1 COS (Devane 2007) for pregnancy in general are already included. |
| 5 | Smith V, Daly D, Lundgren I, Eri T, Benstoem C, Devane D. Salutogenically focused outcomes in systematic reviews of intrapartum interventions: a systematic review of systematic reviews. Midwifery. 2014;30(4):e151-6. |  |

**Stage 2: Studies reporting outcomes for pregnant women with multimorbidity or their children**

| **No** | **Full Reference** | **Study design** | **Country** | **Population** | **Exposure (maternal multimorbidity)** |
| --- | --- | --- | --- | --- | --- |
| 1 | D'Arcy R, Knight M, Mackillop L. A retrospective audit of the socio-demographic characteristics and pregnancy outcomes for all women with multiple medical problems giving birth at a tertiary hospital in the UK in 2016. BJOG: An International Journal of Obstetrics and Gynaecology. 2019;126:128. | Observational, conference abstract | UK | All women giving birth at a tertiary hospital in 2016 | 2 or more medical conditions |
| 2 | Easter SR, Bateman BT, Sweeney VH, Manganaro K, Lassey SC, Gagne JJ, et al. A comorbidity-based screening tool to predict severe maternal morbidity at the time of delivery. American Journal of Obstetrics & Gynecology. 2019;221(3):271.e1-.e10. | Validation | USA | All patients with pregnancies ≥23 weeks gestation presenting for labour and delivery at a single tertiary-care centre from February through July 2018 | Obstetric comorbidity index |
|  | Easter SR, Sweeney V, Manganaro K, Lassey SC, Bateman BT, Robinson JN. 278: Prospective clinical validation of the obstetric comorbidity index for maternal risk assessment. American Journal of Obstetrics and Gynecology. 2019;220:S198-S9. | Validation, conference abstract | USA | All patients with pregnancies at or beyond 23 weeks gestation presenting for labour and delivery from February to June 2018 | Obstetric comorbidity index |
| 3 | Salahuddin M, Mandell DJ, Lakey DL, Eppes CS, Patel DA. Maternal risk factor index and cesarean delivery among women with nulliparous, term, singleton, vertex deliveries, Texas, 2015. Birth. 2019;46(1):182-92. | Observational | USA | Nulliparous, term, singleton, vertex deliveries to women aged 15‐49 years in Texas | Maternal risk factor index (0-4) |
| 4 | Somerville NJ, Nielsen TC, Harvey E, Easter SR, Bateman B, Diop H, et al. Obstetric Comorbidity and Severe Maternal Morbidity Among Massachusetts Delivery Hospitalizations, 1998-2013. Maternal & Child Health Journal. 2019;23(9):1152-8. | Observational | USA | All delivery hospitalizations during 1998–2013 in Massachusetts | Obstetric comorbidity index |
| 5 | Bliddal M, Moller S, Vinter CA, Rubin KH, Gagne JJ, Pottegard A. Validation of a comorbidity index for use in obstetric patients: A nationwide cohort study. Acta Obstetricia et Gynecologica Scandinavica. 2020;99(3):399-405. | Validation | Denmark | All completed pregnancies (both live- and stillborn infants) in Denmark from 1 July 2000 to 1 December 2014 | Obstetric comorbidity index |
| 6 | Brown CC, Adams CE, George KE, Moore JE. Associations Between Comorbidities and Severe Maternal Morbidity. Obstetrics & Gynecology. 2020;136(5):892-901. | Observational | USA | All delivery hospitalisation in year 2016-2017 from the National Inpatient Sample | Number of comorbidities |
| 7 | Cao S, Dong F, Okekpe CC, Dombrovsky I, Valenzuela GJ, Roloff K. Prevalence of the number of pre-gestational diagnoses and trends in the United States in 2006 and 2016. Journal of Maternal Fetal and Neonatal Medicine. 2020. | Observational | USA | All pregnant patients admitted for delivery | Number of pregestational diagnosis |
| 8 | Field CP, Stuebe AM, Verbiest S, Tucker C, Ferrari R, Jonsson-Funk M. 917: Early identification of women likely to be high utilizers of perinatal acute care services. American Journal of Obstetrics and Gynecology. 2020;222:S567-S8. | Observational, conference abstract | USA | Women who received prenatal care and delivered at the North Carolina Women’s Hospital between July 1, 2014, and June 30, 2016, who had at least one prenatal outpatient encounter before 20 weeks’ gestation | Number of non-obstetric diagnosis documented in the first 20 weeks of pregnancy |
| 9 | Fresch R, Stephens KK, DeFranco E. 1193: The combined influence of multiple maternal medical conditions on incidence of primary cesarean section. American Journal of Obstetrics and Gynecology. 2020;222:S734-S5. | Observational, conference abstract | USA | Ohio live birth records from 2006-2015 | Multiple medical comorbidities |
| 10 | Fresch RJ, DeFranco E, Stephen K. The combined influence of maternal medical conditions on the risk of fetal growth restriction. Obstetrics and Gynecology. 2020;135:154S-5S. | Observational, conference abstract | USA | Ohio live birth records from 2006–2015 | Multiple medical comorbidities |
| 11 | Leonard SA, Kennedy CJ, Carmichael SL, Lyell DJ, Main EK. An Expanded Obstetric Comorbidity Scoring System for Predicting Severe Maternal Morbidity. Obstetrics & Gynecology. 2020;136(3):440-9. | Validation | USA | All live births occurring in California-licensed hospitals during 2016 and 2017 | Obstetric comorbidity score |
| 12 | Liu V, Hedderson M, Greenberg M, Kipnis P, Escobar GJ, Ruppel H. Development of an obstetrics comorbidity risk score for clinical and operational use. Journal of Women's Health. 2020;29:A14. | Validation, conference abstract | USA | Pregnancies from Kaiser Permanente Northern California between 2010 and 2017 | Obstetric comorbidity risk score |
| 13 | Main EK, Leonard SA, Menard MK. Association of Maternal Comorbidity With Severe Maternal Morbidity: A Cohort Study of California Mothers Delivering Between 1997 and 2014. Annals of Internal Medicine. 2020;173(11):S11-S8. | Observational | USA | All mothers delivering in California during 1997 to 2014. | Maternal comorbid conditions individually and as a maternal comorbidity index score (Bateman 2013, Easter 2019) |
| 14 | Ranjit A, Olufajo O, Zogg C, Robinson JN, Luo G. To determine if maternal adverse outcomes predicted by obstetric comorbidity index (OBCMI) varies according to race. Obstetrics and Gynecology. 2020;135:37S-8S. | Observational, conference abstract | USA | All admissions for deliveries in the National Inpatient Database (2010–2014) | Obstetric comorbidity index |
| 15 | Salahuddin M, Mandell DJ, Lakey DL, Ramsey PS, Eppes CS, Davidson CM, et al. Maternal comorbidity index and severe maternal morbidity during delivery hospitalizations in Texas, 2011-2014. Birth. 2020;47(1):89-97. | Observational | USA | Delivery‐related hospitalizations among Texan women aged 15‐49 years | Medical (chronic and behavioural) and obstetric (pregnancy‐induced) conditions as measured by the maternal comorbidity index developed by Bateman 2013 |
| 16 | Sutton D, Oberhardt M, Oxford-Horrey CM, Prabhu M, Aubey J, Riley LE, et al. 711 Obstetric comorbidity index corresponds with racial disparity in maternal morbidity providing insight for risk reduction. American Journal of Obstetrics and Gynecology. 2021;224:S445-S6. | Observational, conference abstract | USA | All deliveries in a four-hospital system from January 2016 through January 2020 | Obstetric comorbidity index (Leonard 2020) |
| 17 | Oberhardt M, Sutton D, Oxford-Horrey C, Prabhu M, Sheen JJ, Riley L, et al. 8 Augmenting or Replacing Obstetric Comorbidity Index with Labor & Delivery Features Improves Prediction of Non-Transfusion Severe Maternal Morbidity. American Journal of Obstetrics and Gynecology. 2021;224:S5. | Validation, conference abstract | USA | All women delivering in a four-hospital system from January 2016 through January 2020 | Obstetric comorbidity index (Leonard et al 2020) |
| 18 | Admon LK, Winkelman TNA, Heisler M, Dalton VK. Obstetric Outcomes and Delivery-Related Health Care Utilization and Costs Among Pregnant Women With Multiple Chronic Conditions. Preventing Chronic Disease. 2018;15:E21. | Observational | USA | Deliveries in 2013–2014 from the National Inpatient Sample, a nationally representative sample of hospital discharges in the United State | Multiple chronic conditions |
| 19 | Clapp MA, James KE, Kaimal AJ. The effect of hospital acuity on severe maternal morbidity in high-risk patients. American Journal of Obstetrics & Gynecology. 2018;219(1):111.e1-.e7. | Observational | USA | Hospital deliveries in the 2013 Nationwide Readmission Database | Comorbidity index (Bateman 2013) |
|  | Clapp MA, James KE, Kaimal AJ. The association between hospital acuity and severe maternal morbidity in a nationwide sample. American Journal of Obstetrics and Gynecology. 2018;218:S41-S2. | Observational, conference abstract | USA | Hospital deliveries in the 2013 Nationwide Readmission Database | Comorbidity index (Bateman 2013) |
| 20 | Metcalfe A, Wick J, Ronksley P. Racial disparities in comorbidity and severe maternal morbidity/mortality in the United States: an analysis of temporal trends. Acta Obstetricia et Gynecologica Scandinavica. 2018;97(1):89-96. | Observational | USA | All delivery hospitalizations among women aged 10–55 between 1993 and 2012 recorded in the Nationwide Inpatient Sample data | Comorbidities, individually and as Obstetric comorbidity index score |
| 21 | Aoyama K, D'Souza R, Inada E, Lapinsky SE, Fowler RA. Measurement properties of comorbidity indices in maternal health research: a systematic review. BMC Pregnancy & Childbirth. 2017;17(1):372. | Systematic review | Canada | Pregnant and postpartum women in general wards and intensive care units at acute care hospitals | Comorbidity indices |
| 22 | Cunningham SD, Herrera C, Udo IE, Kozhimannil KB, Barrette E, Magriples U, et al. Maternal Medical Complexity: Impact on Prenatal Health Care Spending among Women at Low Risk for Cesarean Section. Womens Health Issues. 2017;27(5):551-8. | Observational | USA | Women aged 18 to 44 who gave birth in 2011 | Maternal medical complexity including numbers of comorbidities |
| 23 | Hehir MP, Ananth CV, Wright JD, Siddiq Z, D'Alton ME, Friedman AM. Severe maternal morbidity and comorbid risk in hospitals performing <1000 deliveries per year. American Journal of Obstetrics & Gynecology. 2017;216(2):179.e1-.e12. | Observational | USA | Births from 1998 through 2011, from the Nationwide Inpatient Sample | Comorbidity index (Bateman 2013) |
|  | **Additional papers from reference list** |  |  |  |  |
| 24 | Bateman BT, Mhyre JM, Hernandez-Diaz S, Huybrechts KF, Fischer MA, Creanga AA, et al. Development of a comorbidity index for use in obstetric patients. Obstetrics and Gynecology. 2013;122(5):957-65. | Validation | USA | Women who delivered in-hospital and were eligible for Medicaid | Maternal comorbidity index |
| 25 | Metcalfe A, Lix L, Johnson J-A, Currie G, Lyon A, Bernier F, et al. Validation of an obstetric comorbidity index in an external population. BJOG: An International Journal of Obstetrics & Gynaecology. 2015;122(13):1748-55. | Validation | Canada | All women who delivered a live or stillborn infant in a hospital in the Calgary Zone of Alberta Health Services and conceived between 4 November 2007 and 23 February 2008 | Obstetric comorbidity index |
| 26 | Knight M, Bunch K, Tuffnell D, Shakespeare J, Kotnis R, Kenyon S, Kurinczuk JJ (Eds.) on behalf of MBRRACE-UK. Saving Lives, Improving Mothers’ Care - Lessons learned to inform maternity care from the UK and Ireland Confidential Enquiries into Maternal Deaths and Morbidity 2016-18. Oxford: National Perinatal Epidemiology Unit, University of Oxford 2020. | Observational | UK | Women who died during or up to a year after pregnancy in the UK in 2016-18, with pregnant women who had multiple problem as a subgroup | Multiple disadvantage (main elements: mental health, substance use, domestic abuse) |

## Additional File 3c: Studies that were excluded and reasons for exclusion (stage 2)

| **No** | **Full reference** | **Reason for exclusion** |
| --- | --- | --- |
| 1 | Moe HW, Sharma S, Sharma AK. An evaluation of medication appropriateness in pregnant women with coexisting illness in a tertiary care hospital. Perspectives in Clinical Research. 2021;12(1):21-6. | Exposure: not 2 or more long-term conditions that existed before pregnancy |
| 2 | Pluym ID, Tandel M, Kwan L, Mok T, Holliman K, Afshar Y, et al. 57 Randomized Control Trial of Postpartum Visits at 2 weeks and 6 weeks. American journal of obstetrics and gynecology. 2021;224(2):S40‐. | Population: not pregnant women with multimorbidity or their offspring |
| 3 | Little D, Varner C, Park A, Ray J. Emergency department use by pregnant women: A population-based study within a universal healthcare system. Journal of Obstetrics and Gynaecology Canada. 2020;42:680. | Population: not pregnant women with multimorbidity or their offspring. The main purpose was to characterize emergency department visits during pregnancy and number of comorbidities was one of two risk factors examined. |
| 4 | Varner CE, Park AL, Little D, Ray JG. Emergency department use by pregnant women in Ontario: a retrospective population-based cohort study. CMAJ open. 2020;8(2):E304-E12. | Population: not pregnant women with multimorbidity or their offspring. The main aim was to characterise emergency department visits by pregnant women. |
| 5 | McCauley M, Zafar S, van den Broek N. Maternal multimorbidity during pregnancy and after childbirth in women in low- and middle-income countries: a systematic literature review. BMC Pregnancy & Childbirth. 2020;20(1):637. | Outcomes: No types of outcomes that can be extracted. The review reported on the prevalence of physical, psychological and social morbidities (exposures) and the association between these. |
| 6 | Clapp MA, Little SE, Zheng J, Robinson JN, Kaimal AJ. Case mix and the utility of postpartum readmission rates as a marker of quality in obstetrics. American Journal of Obstetrics and Gynecology. 2018;218:S549-S50. | Exposure: not 2 or more long-term conditions that existed before pregnancy. The exposures were listed as individual diseases, the study identified patients based on low / high admission rates hospital then looked for risk factors for readmissions. |
| 7 | Cunningham SD, Magriples U, Thomas JL, Kozhimannil KB, Herrera C, Barrette E, et al. Association Between Maternal Comorbidities and Emergency Department Use Among a National Sample of Commercially Insured Pregnant Women. Academic Emergency Medicine. 2017;24(8):940-7. | Exposure: not 2 or more long-term conditions that existed before pregnancy. The study participants were categorised as one or more comorbidities vs no comorbidities. |

## Additional File 3d: Summary list of extracted outcomes

Pregnancy outcomes were categorised by: (1) maternal or children outcomes and (2) by an established taxonomy of outcomes [68]: mortality/survival, physiological/clinical (further subdivided by pregnancy period of antenatal, peripartum, postnatal and long-term for mothers; fetal, neonatal, infant and longer term for children), life impact/functioning, and resource use. We have also added outcome definitions from The National Maternal and Perinatal Audit (UK)[61].

**Table 1: List of outcomes**

| **No** | **Outcomes** | **Definition of outcomes** | **References** |
| --- | --- | --- | --- |
|  | **MATERNAL OUTCOMES** |  |  |
|  | **Maternal: Mortality / survival** |  |  |
| 1 | Maternal death[4, 15, 16, 33, 40, 54, 55]  Maternal mortality[58]  In hospital maternal mortality[53]  Mortality[18] | From the start of delivery admission to the hospital through 30 days postpartum.*[33]*  Combined with severe maternal morbidity as “severe maternal morbidity and mortality”.[4]  Combined with serious morbidity as “maternal mortality or serious morbidity”.[58]  Combined with end organ injury as a composite outcome of maternal end organ injury or death, during the delivery admission through 30 days postpartum.[52]  Died during or up to six weeks after the end of pregnancy.[15, 54]  Died between six weeks and one year after the end of pregnancy.[54]  Death of a female from any cause related to or aggravated by pregnancy or its management (excluding accidental or incidental causes) during pregnancy and childbirth or within 42 days of pregnancy termination, irrespective of site or duration of the pregnancy.[16]  From core outcome set for multimorbidity[18] | Bliddal 2020[33]  Liu 2020[40]  Admon 2018[4]  Metcalfe 2018[53]  Bateman 2013[52]  Knight 2020[54]  Smith 2018[18]  Devane 2007[15]  Nijagal 2018[16]  Smith 2014[58]  Herman 2021[55] |
|  | **Maternal: Physiological / clinical** |  |  |
|  | ***Antenatal*** |  |  |
| 2 | Pregnancy induced hypertension[35]  Pre-eclampsia[5]  Severe pre-eclampsia[40]  Eclampsia[40]  Intrapartum hypertensive disorders of pregnancy[15]  Hypertensive disorders of/in pregnancy[15, 55]  Postnatal hypertensive disorders of pregnancy[15] | A group of diseases characterized by high blood pressure with or without proteinuria; this group includes pre-eclampsia, eclampsia, and the syndrome of HELLP.[15] | Cao 2020[35]  D’Arcy 2019[5]  Liu 2020[40]  Devane 2007[15]  Herman 2021[55] |
| 3 | Obstetric cholestasis |  | D’Arcy 2019[5] |
| 4 | Gestational diabetes mellitus |  | Cao 2020[35]  Herman 2021[55] |
| 5 | Chorioamnionitis |  | Cao 2020[35] |
| 6 | Fluid abnormalities on ultrasound (oligohydramnios or polyhydramnios) |  | Herman 2021[55] |
| 7 | Oligohydramnios |  | Cao 2020[35] |
| 8 | Polyhydramnios |  | Cao 2020[35] |
| 9 | Placental abruption |  | Cao 2020[35]  Herman 2021[55] |
| 10 | Placental insufficiency |  | Cao 2020[35] |
| 11 | Placenta previa |  | Cao 2020[35] |
| 12 | Smoking rate at booking |  | Bunch 2018[60] |
| 13 | Nausea / vomiting / dehydration |  | Smith 2014[58] |
| 14 | Headache |  | Smith 2014[58] |
|  | ***Peripartum*** |  |  |
| 15 | Types of labour onset | Manner in which labour started, i.e., induced, spontaneous, planned caesarean section. | Devane 2007[15] |
| 16 | Spontaneous rupture of membranes |  | Smith 2014[58] |
| 17 | Preterm premature rupture of membranes |  | Cao 2020[35] |
| 18 | Delivery route[31]  Mode of birth[15]^,^[58] | Vaginal/spontaneous, vaginal/forceps, vaginal/vacuum, and caesarean. Caesarean included those with and without attempted trial of labour.[31]  E.g., spontaneous vaginal, forceps, vaginal breech, caesarean section, vacuum extraction.[15] | Salahuddin 2019[31]  Devane 2007[15]  Smith 2014[58] |
| 19 | Caesarean delivery[4, 37]  Caesarean birth[58, 61]  Caesarean section[55]  Caesarean section delivery rate in Robson group 1 women[60]  Caesarean section delivery rate in Robson group 2 women[60]  Caesarean section delivery rate in Robson group 5 women[60] | Robson group 1: Nulliparous, single cephalic, ≥ 37 weeks, spontaneous labour[60, 62]  Robson group 2: Nulliparous, single cephalic, ≥ 37 weeks, induced or caesarean before labour[60, 62]  Robson group 5: Multiparous, previous caesarean, single cephalic ≥ 37 weeks[60, 62] | Fresch 2020[37]  Admon 2018[4]  Bunch 2018[60]  Smith 2014[58]  Herman 2021[55]  NMPA 2018[61] |
| 20 | Emergency caesarean section |  | D’Arcy 2019[5] |
| 21 | Vaginal birth after previous caesarean section (VBAC)[15]  Trial of labour after previous caesarean delivery[15]  Rate of successful vaginal birth after a single previous caesarean section[60]  Vaginal birth after caesarean section[61] | **NMPA**[61]  **Overall VBAC 2nd birth**: of women having their second baby after having had a caesarean section for their first baby,* the proportion who give birth to their second baby vaginally.  **Attempted VBAC 2nd birth**: of women having their second baby after having had a caesarean section for their first baby, the proportion who attempt to have a vaginal birth for their second baby.  **VBAC 2nd birth in attempted**: of women having their second baby after having had a caesarean section for their first baby and who attempted to have a vaginal birth for their second baby, the proportion who give birth to their second baby vaginally.  * The measure is limited to this group of women because of the limitations of historical records and because this is the largest group of women considering VBAC. The rates reported do therefore not include women who also had a previous vaginal birth. | Devane 2007[15]  Bunch 2018[60]  NMPA 2018[61] |
| 22 | Any instrumental/assisted vaginal birth[58]  Operative vaginal delivery[55]  Instrumental birth[61] | Birth is assisted by the use of an instrument (either ventouse or forceps).[61] | Smith 2014[58]  Herman 2021[55]  NMPA 2018[61] |
| 23 | Normal (i.e., physiological) birth without intervention[15]  Rate of birth without intervention[60]  Birth without intervention[61]  Spontaneous vaginal birth (or ‘normal vaginal birth’) [61]^,^[58] | Vaginal birth without induction, episiotomy, or epidural.[15]  The NMPA *birth without intervention* measure refers to spontaneous birth which starts and progresses spontaneously (i.e. without induction, augmentation with drugs, instrumental or caesarean birth), and without epidural/spinal/general anaesthesia or episiotomy.[61]  The NMPA *spontaneous vaginal birth* measure refers to all vaginal births without the use of instruments. This includes women who have their labour induced, who have augmentation (a ‘drip’ to increase contractions) or an episiotomy.[61] | Devane 2007[15]  Bunch 2018[60]  Smith 2014[58]  NMPA 2018[61] |
| 24 | Place of birth |  | Devane 2007[15] |
| 25 | Anaesthesia with gastric reference (Mendelson's syndrome, etc.) | Mendelson’s syndrome is a chemical pneumonitis due to aspiration of gastric content.[63] | Smith 2014[58] |
| 26 | Use of pharmacological analgesia/anesthesia[15]  Analgesia[58] | E.g., Entonox, epidural, pethidine.[15]  Request for/any type, epidural, narcotics, general anaesthesia (GA), etc.[58] | Devane 2007[15]  Smith 2014[58] |
| 27 | ‘Drugs’ other than analgesics | Administration/ side effects, etc. | Smith 2014[58] |
| 28 | Postnatal administration of drugs |  | Herman 2021[55] |
| 29 | Induction and/or labour augmentation (artificial rupture of membrane/ oxytocin)[58]  Induction of labour[61] | A process by which labour is started artificially, either by giving medications to soften the cervix and start contractions, by a doctor or midwife breaking the waters, or both.[61] | Smith 2014[58]  NMPA 2018[61] |
| 30 | Oxytocin augmentation of labour | Drug used to assist progress of labour. | Devane 2007[15] |
| 31 | Number (count) of pregnancy complications |  | Cao 2020[35] |
| 32 | Adverse event / outcome, serious complication – maternal |  | Smith 2014[58] |
| 33 | Maternal near miss |  | Herman 2021[55] |
| 34 | Procedural or anaesthesia complication |  | Herman 2021[55] |
| 35 | Medication-related serious adverse events | Includes serious allergic reaction and any serious event as a result of medication for e.g., cardiac events, pulmonary embolism and intensive care unit admission. | Herman 2021[55] |
| 36 | Intrapartum haemorrhage[15]  Postpartum haemorrhage[15, 55]  Haemorrhage[35, 40]  Rate of postpartum haemorrhage of ≥1500 ml[60]  Bleeding / blood loss[58]  Obstetric haemorrhage[61] | Excessive blood loss from the birth canal during labour. [15]  Excess blood loss from the birth canal after childbirth.[15]  Of any type and variously defined.[58]  Of women who give birth to a singleton baby between 37+0 and 42+6 weeks of gestation, the proportion who sustained an obstetric haemorrhage of 1500ml or more. Also reported obstetric haemorrhage of 500ml or more.[61] | Cao 2020[35]  Liu 2020[40]  Devane 2007[15]  Bunch 2018[60]  Smith 2014[58]  Herman 2021[55]  NMPA 2018[61] |
| 37 | Infection[35]  Maternal infection[55, 58]  Infectious morbidity[55] | Fever/temperature/sepsis, etc.[58] | Cao 2020[35]  Smith 2014[58]  Herman 2021[55] |
| 38 | Placenta | Retained, manual removal, etc. | Smith 2014[58] |
| 39 | Caesarean section wound infection |  | Devane 2007[15] |
| 40 | Wound[58]  Wound complications[55] | Haematoma, wound healing, fistula of any type, etc.[58] | Smith 2014[58]  Herman 2021[55] |
| 41 | Ruptured uterus[15]  Uterine rupture of dehiscence[55] |  | Devane 2007[15]  Herman 2021[55] |
| 42 | Uterine inversion |  | Herman 2021[55] |
| 43 | Uterine | Expulsive effort, hyperstimulation, rupture, etc. | Smith 2014[58] |
| 44 | Laceration (cervical, vaginal, perineal)  Cervical laceration |  | Cao 2020[35]  Herman 2021[55] |
| 45 | Perineal/vaginal trauma[58]  Perineal trauma[55] | Of any type including episiotomy. | Smith 2014[58]  Herman 2021[55] |
| 46 | Episiotomy |  | NMPA 2018[61] |
| 47 | Intact perineum |  | Smith 2014[58] |
| 48 | Anal sphincter damage[15]  Third‐ and fourth‐degree tear rate among women delivering vaginally[60]  Third and fourth degree tear[61] | “Third degree” (extending into the anal sphincter) and “fourth degree” (anal mucosa) tears. Of women who give birth vaginally to a singleton baby in the cephalic position between 37+0 and 42+6 weeks of gestation, the proportion who sustained a third or fourth degree tear.[61] | Devane 2007[15]  Bunch 2018[60]  NMPA 2018[61] |
| 49 | Pulmonary embolism[15]  Thromboembolic event (deep vein thrombosis, pulmonary embolism)[58]  Venous thromboembolism[55]  Thromboembolism[5] | Also listed under severe maternal morbidity and end organ injury see Tables 2 and 3 | Devane 2007[15]  Smith 2014[58]  Herman 2021[55]  D’Arcy 2019[5] |
| 50 | Transfusion[16]  Blood transfusion[58] | Any transfusion of red blood cells within the first 42 days postpartum[16]  Also listed under severe maternal morbidity and end organ injury see Tables 2 and 3 | Nijagal 2018[16]  Smith 2014[58] |
| 51 | Anaemia | Or any reference to Haemoglobin levels/iron administration.[58] | Smith 2014[58]  Herman 2021[55] |
| 52 | Smoking rate at delivery[60]  Smoking cessation in pregnancy[61] | Of those women who are recorded as being current smokers at their booking visit, the proportion who are no longer smokers by the time of birth.[61] | Bunch 2018[60]  NMPA 2018[61] |
| 53 | Mobility during labour |  | Smith 2014[58] |
| 54 | Pregnancy prolongation |  | Smith 2014[58] |
| 55 | Labour length/duration | Length of any stage, prolonged labour, etc. | Smith 2014[58] |
| 56 | Comfort |  | Smith 2014[58] |
| 57 | Maternal perception of pain experienced  ‘Pain’ | ‘Pain’ of any type including assessment.[58] | Smith 2014[58] |
| 58 | Relaxation |  | Smith 2014[58] |
| 59 | Resuscitation measures, arrest or loss of consciousness |  | Smith 2014[58] |
| 60 | Miscellaneous / other | Fetal-maternal haemorrhage, zavanelli procedure, pulmonary oedema, additional tests, cord prolapse, etc. | Smith 2014[58] |
| 61 | Blood pressure |  | Smith 2014[58] |
| 62 | Surgical reference[58]  Additional operations[55] | Type of surgery, duration of surgery, etc. [58] | Smith 2014[58]  Herman 2021[55] |
| 63 | Dilation and curettage for retained products of conception |  | Herman 2021[55] |
| 64 | Extension of uterine incision |  | Herman 2021[55] |
| 65 | Symphysiotomy |  | Smith 2014[58] |
| 66 | Hysterectomy | Also defined in severe maternal morbidity, see Table 2. | Herman 2021[55] |
| 67 | Respiratory morbidity | Also defined in severe maternal morbidity and end organ injury, see Table 2 and 3. | Herman 2021[55] |
| 68 | Renal impairment | Also defined in severe maternal morbidity and end organ injury, see Table 2 and 3. | Herman 2021[55] |
| 69 | Tissue injury (bladder and/or bowel injury) | Also defined in severe maternal morbidity, see Table 2. | Herman 2021[55] |
| 70 | Coagulation abnormalities  Coagulopathy | Also defined in severe maternal morbidity and end organ injury, see Table 2 and 3. | Herman 2021[55] |
| 71 | Hepatic complications | Also defined in end organ injury, see Table 3. | Herman 2021[55] |
| 72 | Cardiac complications | Also defined in severe maternal morbidity, see Table 2. | Herman 2021[55] |
| 73 | Bowel obstruction |  | Herman 2021[55] |
| 74 | Pulmonary oedema | Also defined in severe maternal morbidity and end organ injury, see Table 2 and 3. | Herman 2021[55] |
| 75 | Abnormal maternal biomarkers |  | Herman 2021[55] |
| 76 | Severe maternal morbidity (SMM) | See Table 2 and Table 3 | Easter 2019[29]  Sommerville 2019[32]  Brown 2020[34]  Field 2020[36]  Leonard 2020[39]  Main 2020[41]  Ranjit 2020[42]  Salahuddin 2020[43]  Admon 2018[4]  Clapp 2018[47]  Hehir 2017[51]  Smith 2014[58] |
| 77 | Non transfusion SMM | Severe maternal morbidity excluding deliveries that had an indicator of blood transfusion but no other severe maternal morbidity indicator.*[34]* | Oberhardt 2021[45]  Brown 2020[34]  Leonard 2020[39]  Sutton 2020[44] |
| 78 | End organ injury[33, 52]  End organ damage[53] | See Table 3 | Bliddal 2020[33]  Bateman 2013[52]  Metcalfe 2015[53] |
|  | ***Postnatal and long-term (beyond birth episode)*** |  |  |
| 79 | Postnatal depression[15]  Postpartum depression[16] | Assessed via the Patient Health Questionnaire-2 (PHQ-2) with optional follow-up with the Edinburgh Postnatal Depression Scale (EPDS).[16, 64-66] | Devane 2007[15]  Nijagal 2018[16] |
| 80 | Puerperal psychosis |  | Devane 2007[15] |
|  | **Maternal: Life impact / functioning** |  |  |
| 81 | Maternal fecal incontinence[15]  Incontinence[16, 58] | Tracked via either the ICIQ-SF or Wexner.[16]  Any type.[58] | Devane 2007[15]  Nijagal 2018[16]  Smith 2014[58] |
| 82 | Pain with intercourse | Tracked via PROMIS SFFAC102 PR. | Nijagal 2018[16] |
| 83 | Health-related quality of life[16, 18] | From core outcome set for multimorbidity.[18]  Tracked via the Patient-Reported Outcomes Measurement Information System (PROMIS) Global10.[16] | Smith 2018[18]  Nijagal 2018[16] |
| 84 | Well-being[58] | Mother/father, psychological/emotional.[58] | Smith 2014[58] |
| 85 | Mental health[18]  Maternal negative related expression[58] | From core outcome set for multimorbidity[18]  Anxiety, dissatisfaction, fatigue, depression, low self- esteem, post traumatic stress disorder, etc.[58] | Smith 2018[18]  Smith 2014[58] |
| 86 | Treatment burden | From core outcome set for multimorbidity | Smith 2018[18] |
| 87 | Self-rated health | From core outcome set for multimorbidity | Smith 2018[18] |
| 88 | Self-management behaviour | From core outcome set for multimorbidity | Smith 2018[18] |
| 89 | Self-efficacy | From core outcome set for multimorbidity | Smith 2018[18] |
| 90 | Perceived/personal control |  | Smith 2014[58] |
| 91 | Adherence | From core outcome set for multimorbidity | Smith 2018[18] |
| 92 | Activities of daily living | From core outcome set for multimorbidity | Smith 2018[18] |
| 93 | Physical function | From core outcome set for multimorbidity | Smith 2018[18] |
| 94 | Physical activity | From core outcome set for multimorbidity | Smith 2018[18] |
| 95 | Mother-infant attachment[16]  Positive relationship with infant/bonding[58]  Negative expression of mother–infant interaction (detachment, difficulty with infant, prolonged crying, etc.)[58] | Tracked via the Mother-Infant  Bonding Scale (MIBS). [16] | Nijagal 2018[16]  Smith 2014[58] |
| 96 | Confidence with role as a mother[16]  Maternal parenting confidence[58] | How confident [will you feel when your baby is born/do you feel about looking after your baby]? Not at all confident/Not very confident/Somewhat confident/ Confident/Very confident.[16] | Nijagal 2018[16]  Smith 2014[58] |
| 97 | Care giver experience/satisfaction |  | Smith 2014[58] |
| 98 | Views (mother's and/or father's) |  | Smith 2014[58] |
|  | **Maternal: Resource use** |  |  |
| 99 | Health care use | From core outcome set for multimorbidity.[18] | Smith 2018[18] |
| 100 | Late maternal complication | Admission or re-admission within the first 42 days postpartum for childbirth related complications | Nijagal 2018[16] |
| 101 | High utilisation of perinatal acute care services | Three or more obstetric triage, emergency department or inpatient admissions during pregnancy. | Field 2020[36] |
| 102 | Unscheduled visit to the emergency department or clinic |  | Herman 2021[55] |
| 103 | Hospitalisation[58]  Need for hospital admission[55] | Length of stay, admission, readmission, etc.[58] | Smith 2014[58]  Herman 2021[55] |
| 104 | Emergency department visit | 90 days following birth. | Field 2020[36] |
| 105 | Readmission[36]  Maternal postnatal readmission to hospital[15]  Unplanned maternal readmission[61] | 90 days following birth.[36]  Of women giving birth to a singleton baby between 37+0 and 42+6 weeks of gestation, those who have an unplanned, overnight readmission to hospital within 42 days of giving birth, excluding those accompanying an unwell baby.[61] | Field 2020[36]  Devane 2007[15]  NMPA 2018[61] |
| 106 | High dependency unit/postnatal stay |  | Herman 2021[55] |
| 107 | Maternal intensive care unit (ICU) admission[52, 55, 58]  Mother requires admission to intensive care[15]  Maternal need for intensive care[16] | During the delivery hospitalization through 30 days postpartum.^[52]^  Admission to an ICU or a unit that provides 24-h medical supervision and is able to provide mechanical ventilation or continuous vasoactive drug support at any point during pregnancy through 42 days postpartum for pregnancy or childbirth related complications.[16] | Bateman 2013[52]  Devane 2007[15]  Nijagal 2018[16]  Smith 2014[58]  Herman 2021[55] |
| 108 | Need for hospital transfer | Health care utilisation. | Admon 2018[4] |
| 109 | Hospital length of stay[4]  Maternal length of stay[16]  Extended length of stay for delivery[53] | Health care utilisation.[4]  Number of consecutive days in the hospital from delivery to discharge.[16]  A length of stay ≥3 days following a vaginal delivery or ≥5 days following a caesarean delivery.[53] | Admon 2018[4]  Nijagal 2018[16]  Metcalfe 2015[53] |
| 110 | Health care cost[4, 18]  Health care expenditure during pregnancy[50]  Cost / economic outcomes[58] | Delivery-associated hospital charges. Mean charges and cost per delivery hospitalisation.[4]  Health care expenditure for the entire pregnancy, prenatal and childbirth periods. The prenatal observation period for each patient was defined as date of admission for childbirth minus 300 days.^[50]^  From core outcome set for multimorbidity, so not limited to pregnancy period only.[18] | Admon 2018[4]  Cunningham 2017[50]  Smith 2018[18]  Smith 2014[58] |
| 111 | Communication | Consultation related, from core outcome set for multimorbidity. | Smith 2018[18] |
| 112 | Shared decision making[18]  Confidence as an active participant in healthcare decisions[16] | Consultation related, from core outcome set for multimorbidity.[18]  Thinking about your care during [your pregnancy/your labour and birth/the months after your baby was born], were you given information about your choices for maternity care? Were you given enough information to help you decide about your care? Were you given information at the right time to help you decide about your care? No/To some extent/Yes.[16] | Smith 2018[18]  Nijagal 2018[16] |
| 113 | Prioritization | Consultation related, from core outcome set for multimorbidity. | Smith 2018[18] |
| 114 | Quality health care (patient-rated)[18]  Maternal satisfaction (antenatal)[15]  Maternal satisfaction (intrapartum)[15]  Maternal satisfaction (postnatal)[15]  Satisfaction with the results of care[16]  Maternal satisfaction with care experience[58]  Confidence in healthcare providers[16] | From core outcome set for multimorbidity.[18]  How satisfied are you with the results of your care during [your pregnancy/your labour and birth/the months after your baby was born]? Very unsatisfied/Unsatisfied/Neither satisfied nor dissatisfied/Satisfied/Very satisfied.[16]  Do you have confidence and trust in the staff caring for you? No/To some extent/Yes.[16] | Smith 2018[18]  Devane 2007[15]  Nijagal 2018[16]  Smith 2014[58] |
| 115 | Birth experience | Assessed via the The Birth Satisfaction Scale - Revised.[16, 67] | Nijagal 2018[16] |
|  | **OUTCOMES FOR CHILDREN** |  |  |
|  | **Children: Mortality / survival** |  |  |
| 116 | Intrauterine fetal demise[35]  Fetal death[58] |  | Cao 2020[35]  Smith 2014[58] |
| 117 | Stillbirth | A fetal death in late pregnancy.[15]  Pregnancy loss at or after 28 + 0 weeks gestation of a birth weight of greater or equal to 1000 g.[16] | Devane 2007[15]  Nijagal 2018[16]  Smith 2014[58] |
| 118 | Neonatal death[15, 16]  Neonatal loss[58] | Death before the age of 28 completed days after live birth.[15]  Death of a live born neonate up to 28 days of life.[16] | Devane 2007[15]  Nijagal 2018[16]  Smith 2014[58] |
| 119 | Perinatal death |  | Herman 2021[55] |
|  | **Children: Physiological / clinical** |  |  |
|  | ***Fetal*** |  |  |
| 120 | Intrauterine growth restriction[15, 35]  Fetal growth restriction (FGR)[38]  Severe fetal growth restriction (SFGR)[38] | Commonly used when the birthweight is at or below the 10th percentile for gestational age and sex.[15]  FGR: birthweight 10th percentile for gestational age[38]  SFGR: birthweight 5th percentile for gestational age[38] | Cao 2020[35]  Fresch 2020[38]  Devane 2007[15] |
| 121 | Non-reassuring fetal heart tones[35]  Foetal heart rate changes necessitating delivery[55] |  | Cao 2020[35]  Herman 2021[55] |
| 122 | Foetal heart rate monitoring |  | Smith 2014[58] |
| 123 | Foetal blood sampling | Umbilical cord blood, FBS, lactate. | Smith 2014[58] |
| 124 | Foetal position (malpresentation, change, etc.)[58]  Malpresentation[35, 55] |  | Smith 2014[58]  Cao 2020[35]  Herman 2021[55] |
| 125 | Ultrasound sign |  | Herman 2021[55] |
| 126 | Abnormal doppler findings on ultrasound |  | Herman 2021[55] |
|  | ***Neonatal (first 28 days)*** |  |  |
| 127 | Preterm delivery[4, 5, 35]  Preterm labour[15]  Spontaneous preterm birth[16]  Iatrogenic preterm birth[16]  Preterm[58]  Preterm birth[55] | < 37 weeks[4]  Onset of labour before 37 completed weeks of pregnancy.[15]  Live birth at < 37 +0 weeks gestation occurring after spontaneous labour or rupture of membranes.[16]  Caesarean or labour induction before < 37 weeks + 0 gestation excluding those occurring after spontaneous labour or rupture of membranes.[16]  Birth, retinopathy of prematurity, gestational age at birth.[58] | D’Arcy 2019[5]  Cao 2020[35]  Admon 2018[4]  Devane 2007[15]  Nijagal 2018[16]  Smith 2014[58]  Herman 2021[55] |
| 128 | Prematurity |  | Herman 2021[55] |
| 129 | Apgar score[58]  Apgar score at 5 min[15, 61]  Proportion of babies born at term with an Apgar score <7 at 5 minutes[60]  Low Apgar score[55] | At 1, 5 or 10 minutes or <7 or ‘low’ at ≤5 minutes.[58]  Of liveborn, singleton babies born between 37+0 and 42+6 weeks of gestation, the proportion who are assigned an Apgar score of less than 7 at five minutes of age.[61] | Devane 2007[15]  Bunch 2018[60]  Smith 2014[58]  Herman 2021[55]  NMPA 2018[61] |
| 130 | Gestational age at birth[15] |  | Devane 2007[15] |
| 131 | Infant birthweight[15]  Birthweight[58]  Birth weight abnormalities (including small and large for gestational age)[55] |  | Devane 2007[15]  Smith 2014[58]  Herman 2021[55] |
| 132 | Small for gestational age[61] | Term babies with a birth weight below the 10th centile, and below the 2nd centile using UK 1990 charts.[61] | NMPA 2018[61] |
| 133 | Low birth weight |  | D’Arcy 2019[5] |
| 134 | Large for gestational age |  | Cao 2020[35] |
| 135 | Meconium[35]  Meconium aspiration[15]  Meconium-stained liquor / meconium aspiration syndrome[58] | The newborn inhales a mixture of meconium and amniotic fluid, either in the uterus or just after delivery.[15] | Cao 2020[35]  Devane 2007[15]  Smith 2014[58] |
| 136 | Neonatal resuscitation required[15]  Resuscitation measures, arrest or loss of consciousness[58] |  | Devane 2007[15]  Smith 2014[58] |
| 137 | Oxygen dependence | Administration of oxygen by any route for greater than 24 hours at any point during the first 28 days of life. | Nijagal 2018[16] |
| 138 | Neonatal respiratory morbidity |  | Herman 2021[55] |
| 139 | Birth asphyxia[15]  Asphyxia or acidaemia[55] | Occurs when a baby does not receive enough oxygen before, during, or just after birth.[15] | Devane 2007[15]  Herman 2021[55] |
| 140 | Any pH levels <7.20 and BD >12.0 |  | Smith 2014[58] |
| 141 | Hypoxic ischemic encephalopathy | A condition of injury to the brain.[15] | Devane 2007[15]  Herman 2021[55] |
| 142 | Babies with encephalopathy[61] | The proportion of singleton babies born at 35+0 to 42+6 weeks of gestation with encephalopathy in the first 72 hours of life, defined as showing two or more of the following neurological signs in the same day within the first 72 hours of life:  - abnormal tone  - reduced consciousness (lethargic or comatose)  - convulsions (seizures).[61] | NMPA 2018[61] |
| 143 | Intraventricular haemorrhage |  | Herman 2021[55] |
| 144 | Periventricular leukomalacia |  | Herman 2021[55] |
| 145 | Retinopathy of prematurity |  | Herman 2021[55] |
| 146 | Neonatal fitting/seizures[15]  Seizures[55] |  | Devane 2007[15]  Herman 2021[55] |
| 147 | Congenital anomaly | Chromosomal, genetic, and/or structural.[15] | Devane 2007[15]  Herman 2021[55] |
| 148 | Patent ductus arteriosus |  | Herman 2021[55] |
| 149 | Neonatal infection[15, 58]  Neonatal sepsis[55]  Infectious morbidity[55] | Fever/sepsis including specific types of infections.[58] | Devane 2007[15]  Smith 2014[58]  Herman 2021[55] |
| 150 | Shoulder dystocia |  | Devane 2007[15] |
| 151 | Jaundice[58]  Hyperbilirubinaemia[55] |  | Smith 2014[58]  Herman 2021[55] |
| 152 | Transition to extra-uterine life |  | Smith 2014[58] |
| 153 | Necrotizing enterocolitis / bowel perforation |  | Herman 2021[55] |
| 154 | Infant requiring intubation[15]  Intubation /ventilation[55]  Babies receiving mechanical ventilation[61] | Mechanical ventilation refers to invasive ventilation with an endotracheal tube and ventilator. Therefore, babies requiring non-invasive breathing support such as CPAP (continuous positive airway pressure) are not included in this measure. The time frame for this measure is limited to the first 72 hours of life in order to reflect morbidity that is more likely to be attributed to events around the time of birth. Of liveborn, singleton babies born between 37+0 and 42+6 weeks of gestation, the proportion who receive mechanical ventilation in the first 72 hours of life.[61] | Devane 2007[15]  Herman 2021[55]  NMPA 2018[61] |
| 155 | Hypoglycaemia |  | Herman 2021[55] |
| 156 | Foetal or neonatal anaemia |  | Herman 2021[55] |
| 157 | Inotropic support / hypotension |  | Herman 2021[55] |
| 158 | Birth injury to infant[15]  Birth injury[16]  Neonatal birth trauma[55]  Labour and/or birth trauma[58] | Subdural and cerebral haemorrhage, massive epicranial subaponeurotic haemorrhage, other injuries to skeleton due to birth trauma, injury to spine and spinal cord due to birth trauma, injury to brachial plexus due to birth trauma, other cranial and peripheral nerve injuries due to birth trauma in single live-born neonates.[16] | Devane 2007[15]  Nijagal 2018[16]  Herman 2021[55]  Smith 2014[58] |
| 159 | Peripheral nerve injury (at discharge from hospital) |  | Herman 2021[55] |
| 160 | Basal skull fracture |  | Herman 2021[55] |
| 161 | Spinal cord injury |  | Herman 2021[55] |
| 162 | Hypothermia |  | Herman 2021[55] |
| 163 | Decreased response to pain |  | Herman 2021[55] |
| 164 | Stupor |  | Herman 2021[55] |
| 165 | Clinically significant genital injury |  | Herman 2021[55] |
| 166 | Hypotonia |  | Herman 2021[55] |
| 167 | Coma |  | Herman 2021[55] |
| 168 | Tube feeding |  | Herman 2021[55] |
| 169 | Loss to follow-up |  | Herman 2021[55] |
| 170 | Ischemic injury |  | Herman 2021[55] |
| 171 | Amniotic band syndrome |  | Herman 2021[55] |
| 172 | Twin anaemia-polycythaemia sequence (TAPS) |  | Herman 2021[55] |
| 173 | Twin-to-twin transfusion syndrome (TTS) reoccurrence |  | Herman 2021[55] |
| 174 | Systemic inflammatory response syndrome |  | Herman 2021[55] |
| 175 | Allergic reaction |  | Herman 2021[55] |
| 176 | Postnatal administration of drugs |  | Herman 2021[55] |
| 177 | Composite of infant morbidity outcomes | Short-/long-term; including any disability, hypoxic ischemic encephalopathy (HIE), asphyxia, seizures, respiratory distress syndrome (RDS), Periventricular leukomalacia (PVL), cerebral palsy, etc. | Smith 2014[58] |
| 178 | Skin to skin contact[61] | Of liveborn babies born between 34+0 and 42+6 weeks of gestation, the proportion who received skin to skin contact within one hour of birth.[61] | NMPA 2018[61] |
|  | ***Infant (first 1 year)*** |  |  |
| 179 | Method of infant feeding[15]  Breastfeeding at discharge[15]  Breastfeeding initiation rate[60]  Breastfeeding rate at 6–8 weeks[60]  Breastfeeding at 3 months[15]  Success with breastfeeding[16]  Confidence with breastfeeding[16]  Breastfeeding[58]  Negative expression of breastfeeding (failure, not established, etc.)[58]  Babies receiving breast milk[61] | (Success) Please indicate how you are feeding your baby. My baby has received only breast milk in the past 7 days. This may include breast milk in a bottle/My baby has received a combination of breast milk, formula, or water in the past 7 days/My baby has received only formula, water, or other liquids but not breast milk in the past 7 days.[16]  (Confidence) How confident do you feel about breastfeeding? Not at all confident/Not very confident/ Somewhat confident/Confident/Very confident. Option to track via the Breastfeeding Self-Efficacy Scale – Short Form (BSES-SF).[16]  E.g., initiation, duration, success[58]  Of liveborn babies born between 34+0 and 42+6 weeks of gestation, the proportion who received any breast milk for their first feed, and the proportion receiving any breast milk at the time of discharge from the maternity unit.[61] | Devane 2007[15]  Nijagal 2018[16]  Bunch 2018[60]  Smith 2014[58]  NMPA 2018[61] |
|  | ***Longer term (prepubertal 2-11 years old, pubertal 12-18 years old, adulthood)*** |  |  |
| 180 | Abnormal neurodevelopmental outcome at age 2 years |  | Herman 2021[55] |
| 181 | Severe cerebral palsy at 2 years corrected age for prematurity |  | Herman 2021[55] |
|  | **Children: Resource use** |  |  |
| 182 | Admission to newborn critical care unit[36]  Neonatal admission to special care and/or intensive care unit[15]  Neonatal admission to NICU/SCBU[58]  Neonatal intensive care unit admission[55]  Proportion of babies born at term admitted to the neonatal intensive care unit[60]  Babies admitted to a neonatal unit[61] | **NMPA**[61]  Term: of liveborn, singleton babies born between 37+0 and 42+6 weeks of gestation, the proportion who are admitted to a neonatal unit.  Late preterm: of liveborn, singleton babies born between 34+0 and 36+6 weeks of gestation, the proportion who are admitted to a neonatal unit. | Field 2020[36]  Devane 2007[15]  Bunch 2018[60]  Smith 2014[58]  Herman 2021[55]  NMPA 2018[61] |
| 183 | Neonate length of stay | Number of consecutive days in hospital from birth through 28 days of life. | Nijagal 2018[16] |
| 184 | Neonatal readmission to hospital[15]  Proportion of babies readmitted to hospital at <30 days of age[60] |  | Devane 2007[15]  Bunch 2018[60] |
| 185 | Transfer to long-term care facility |  | Herman 2021[55] |

**Table 2: Severe maternal morbidity**

| **No** | **List of morbidities under severe maternal morbidity (SMM)** | **SMM definition** | **Paper that the SMM was extracted from** |
| --- | --- | --- | --- |
| 1 | 1. Haemorrhage 2. Hypertension / neurologic 3. Renal 4. Sepsis 5. Pulmonary 6. Cardiac 7. ICU/invasive monitoring 8. Surgical/bladder/bowel complications 9. Anaesthesia complications | Based on the American College of Obstetrician Gynaecologists and Society for Maternal-Fetal Medicine consensus definition. These guidelines define SMM as **unintended outcomes** of the process of labour and delivery that **result in significant short-term or long-term consequences** to a woman’s health. The consensus statement specifically avoids providing a comprehensive list of outcomes to define SMM but propose potential scenarios that constitute this outcome and classify SMM into 9 different causes.[29] | Easter 2019[29] |
| 2 | 1. Acute myocardial infarction 2. Acute renal failure 3. Adult respiratory distress syndrome 4. Amniotic fluid embolism 5. Aneurysm 6. Blood transfusion[53] 7. Cardiac arrest/ventricular fibrillation 8. Disseminated intravascular coagulation 9. Eclampsia 10. Heart failure during procedure or surgery 11. Internal injuries of thorax, abdomen, and pelvis 12. Intracranial injuries 13. Puerperal cerebrovascular disorders 14. Pulmonary oedema / acute heart failure 15. Severe anaesthesia complications 16. Sepsis 17. Shock 18. Sickle cell anaemia with crisis 19. Thrombotic embolism[32] / air and thrombotic embolsim[48] 20. Cardiac monitoring 21. Conversion of cardiac rhythm 22. Hysterectomy 23. Operations of the heart and pericardium 24. Temporary tracheostomy 25. Ventilation | Lifesaving procedures or life-threatening events from delivery hospitalization administrative data.[32] | Sommerville 2019[32]  Metcalfe 2018[48] |
| 3 | **Diagnosis-based indicators**   1. Acute myocardial infarction 2. Aneurysm 3. Acute Renal Failure 4. Adult respiratory distress syndrome 5. Amniotic fluid embolism 6. Cardiac arrest or ventricular fibrillation 7. Disseminated intravascular coagulation 8. Eclampsia 9. Heart failure or arrest during surgery or procedure 10. Puerperal cerebrovascular disorders 11. Pulmonary oedema or Acute heart failure 12. Severe anaesthesia complications 13. Sepsis 14. Shock 15. Sickle cell disease with crisis 16. Air and thrombotic embolism   **Procedure-based indicators**   1. Conversion of cardiac rhythm 2. Blood products transfusion 3. Hysterectomy 4. Temporary tracheostomy 5. Ventilation | Identified using the Centres for Disease Control and Prevention (CDC) SMM indicator list.[34, 39]  SMM defined as 1) had at least one of the five procedure-based indicators or 2) had at least one of the 16 diagnosis-based indicators and additionally had: a) in hospital death, b) a caesarean delivery with a length of stay 5 days or longer, or c) a vaginal delivery with a length of stay 3 days or longer.[34]  The Alliance for Innovation on Maternal Health classification scheme was followed for defining SMM, where delivery hospitalizations with any of the 21 conditions were defined as delivery hospitalizations with SMM.[43]  Combined with severe maternal morbidity as “severe maternal morbidity and mortality”.[4] | Brown 2020[34]  Leonard 2020[39]  Main 2020[41]  Salahuddin 2020[43]  Field 2020[36]  Admon 2018[4] |

SMM: Severe maternal morbidity

**Table 3: End organ injury**

| **No** | **List of end organ injury** | **Definition** | **Paper that the end organ injury was extracted from** |
| --- | --- | --- | --- |
| 1 | 1. Acute Heart Failure 2. Acute Liver Disease 3. Acute Myocardial Infarction 4. Acute Renal Failure 5. Acute Respiratory Distress Syndrome/Respiratory Failure 6. Coma 7. Delirium 8. Disseminated Intravascular Coagulation/Coagulopathy 9. Puerperal Cerebrovascular Disorders 10. Pulmonary Oedema 11. Pulmonary Embolism 12. Sepsis 13. Shock 14. Status Asthmaticus 15. Status Epilepticus | End organ injury from the start of delivery admission to the hospital through 30 days postpartum, from Bateman 2013.[33, 52]  Outcome was named as “Severe Maternal Morbidity” in the study.[47, 51]  The presence of any 1 of 15 diagnoses representative of acute organ injury and critical illness.[51]  Combined with death as a composite outcome of maternal end organ injury or death, during the delivery admission through 30 days postpartum.[52] | Bliddal 2020[33]  Clapp 2018[47]  Hehir 2017[51]  Bateman 2013[52]  Metcalfe 2015[53] |
