# Supplementary material for: The development of a core outcome set for studies of pregnant women with multimorbidity
Source: BMC Med. 2023 Aug 21;21:314. doi: 10.1186/s12916-023-03013-3 (PMC10441728; doi:10.1186/s12916-023-03013-3)
Supplement: Supplementary file 6 — Additional file 6. First consensus meeting report. [file 12916_2023_3013_MOESM6_ESM.docx]

## Additional File 6: First consensus meeting report

**First consensus meeting: Core outcome set for studies of pregnant women with multiple long-term conditions (multimorbidity)**

**Date:** 12^th^ Sept 2022

**Time:** 1130am to 1330pm UK time

**Aim of the meeting**

To determine whether outcomes for *Further discussion* should be included for discussion in the second consensus meeting, in addition to those that have already reached *Consensus in* in the Delphi surveys. Outcomes were eligible for *Further discussion* if in the second survey: (i) ≥70% of all participants rated the outcome as *Important but not critical,* or (ii) when ≥70% of participants in one stakeholder group rated an outcome as *Critically important* but *Consensus in* was not reached.

**Premeeting task**

Participants were sent the list of *Borderline* outcomes with plain English explanation in advance. They were asked to consider whether these outcomes should be included ahead of the meeting.

**Summary of round robin and group discussions for the 15 outcomes for *Further discussion***

**1. Maternal outcomes: Miscarriage**

Women with multiple long-term conditions and on medications may be at higher risk of miscarriage and have taken a lot of preconception preparation such as medication adjustment. This is the only marker for fertility. The outcome has huge psychological impact, intervention may improve this outcome. Potentially covered by *Death before birth* already. Need to be able to clearly distinguish whether the miscarriage is attributable to the women’s condition.

**2. Maternal outcomes: Termination of pregnancy**

Potentially covered by *Death before birth* already. Needs to be more specific as there is a wide range of reasons for termination of pregnancy (TOP). Important to distinguish between TOP because of medical reasons where the survival of mother and child is at risk, social reasons, lack of support, and whether women were being coerced. More important to measure whether women or couples received the right kind of care around the decision.

**3. Maternal outcomes: Gestational diabetes**

For: This outcome is important in certain long-term conditions. For women with multiple long-term conditions, having a new condition to manage in pregnancy, new set of treatments and appointments can be challenging.

Against: In low middle income countries, women with these conditions tend to progress to chronic conditions. Many agreed that there are already a lot of studies covering these outcomes and hence should not be in the core outcome set. Discussed whether these outcomes would already be included in multimorbidity (exposure state, but currently this is limited to pre-existing health conditions), or already included in the outcome *Development of new long-term conditions* as women with these conditions should receive lifelong monitoring.

**4. Maternal outcomes: Gestational hypertension**

This outcome was discussed together with *gestational diabetes*, so please see above.

**5. Maternal outcomes: Involvement in care decisions for types of birth**

Birth experience can have long-term impact. Women with multiple long-term conditions may need different types of birth. Discussed about the possibility of combining this outcome with *Involvement with care decision in overall care*, and list specific decisions as subdomains of the outcome. Women spoke about concerns that combining this outcome with *Overcall care* would make the outcome too broad and very subjective. Important to monitor the two aspects of involvement in care decisions (types of birth and pain relief), whether pregnant women with multiple long-term conditions were abused, discriminated against or consented properly, as these outcomes can lead to post-traumatic stress disorder and postpartum mental illness. Highlighted evidence that appalling treatment of women is still a problem now based on a recent United Nation report and the Ockendon report.

**6. Maternal outcomes: Involvement in care decisions for pain relief**

Types of pain relief is already a major focus in antenatal care provision and there is alot of information provision. Discussed about not being able to access the care or pain relief due to circumstances, despite women being involved in the care decisions and agreeing on a pain relief care plan antenatally. Hence it is important to consider *Experience of care*, not just *Involvement in care decisions*, and to consider whether *Involvement in care decisions* can be aligned with *Experience of care.* Pain management is important for women with chronic pain even before labour. People with mental illness or other health conditions may be discriminated against and denied pain relief even in non-labour context.

**7. Maternal outcomes: Shoulder dystocia**

Discussed whether should exclude given this outcome is rare and obstetric teams are usually very well prepared to manage it. However, when it happens, the consequences for mother and baby can be serious. If women with multiple long-term conditions are usually advised to have a caesarean birth for medical reasons, then this risk may not be relevant.

**8. Maternal outcomes: Quality & experience of care**

Prioritised by clinicians as want women and babies to be safe, well and have a positive experience. If negative experiences are not addressed, women may develop fear of childbirth in the future. Very few outcomes in the current core outcome set cover subjective experiences, many are hard clinical outcomes. There are ways to collect subjective experiences nowadays. The experiences will differ based on the women’s long-term conditions.

To consider whether this outcome can be combined with *Involvement in care*, as being involved in care planning does not mean the care delivered was what was agreed on, and those who were not involved in their care decisions would usually not report a good experience of care.

**9. Maternal outcomes: Care for long-term conditions**

Women get good care for some long-term conditions, such as diabetes and lupus, but not for their other conditions, care is fragmented, so putting it all together is very important.

**10. Children’s outcomes: Separation of mother from newborn baby**

Potentially have impact on other areas such as feeding, bonding, longer term mental health and psychological outcomes if separated for long periods of time. However, this outcome is also well covered by the outcome *Admission to neonatal unit*.

**11. Children’s outcomes: Blood transfusion**

Key reasons for blood transfusion in the neonatal period are baby’s conditions at birth (related to problems at birth) and prematurity. There are already other outcomes that would cover baby’s condition at birth and prematurity: gestational age at birth, birthweight, APGAR score. Therefore, even though this outcome is important, would not include in the core outcome set.

**12. Children’s outcomes: Inheritance of mothers’ condition**

This outcome needs to be more specific in terms of inheritance of what types of health conditions, e.g., genetic conditions such as Down syndrome, haemophilia, sickle cell. Need to differentiate from environmental factors. Need to consider if individual long-term conditions may already have good evidence available for inheritability and not duplicate the work.

For: Knowing the risk, mothers can then watch out for early signs of the condition in their children and take mitigating actions.

Against: Some conditions may manifest later in life. Risk of inheritance of health conditions already discussed quite a lot before conception, unsure if care can impact on whether baby inherits the condition.

**13. Children’s outcomes: Quality of life**

Currently there is no good measure for babies’ quality of life, but including it in the core outcome set may encourage researchers to strive to do so in the future. Important as a long-term outcome for children. Can also impact on mother’s quality of life and mother’s perceived well-being.

**14. Children’s outcomes: General cognitive ability**

For: It is problematic to combine a wide range of different functions (general gross motor ability, general fine motor ability, general cognitive ability etc) with different severity into one binary outcome under *Neurodevelopmental conditions*. *General cognitive ability* is how children will do in school in terms of their thinking. It is not well covered by the broad heading *Neurodevelopmental conditions.* There was strong feedback from parents in the neonatal units that they want to know the impact of things that happened on the neonatal unit and during pregnancy on the development of the child, so they can make decisions on treatments for the baby and care the mother receives during pregnancy. Women would want to know the impact of medication during pregnancy on children’s cognitive ability.

Against: Already covered by some other outcomes, e.g. *Brain injury* that is directly related to something going wrong within birth or pregnancy. Concerned about this outcome being misused and lead to ableism and eugenics.

**15. Children’s outcomes: General gross motor ability**

Already covered by some other outcomes such as *Cerebral Palsy.*

**Other general comments**

| **Comments** | **Response** |
| --- | --- |
| (1) Mother’s quality of life is not included in the core outcome set. | This outcome did not reach *Consensus in* in the second Delphi survey.  Percentage of participants that rated this outcome as *Critically important:*  - All participants 59.48%  - Women / partner 64.10%  - Health professionals / researchers 57.14% |
| (2) There were more health professionals / researchers stakeholders compared to women stakeholders at the consensus meeting. Women’s point of view should be prioritised. | We made a post hoc decision to address this by also including outcomes that were voted in by women stakeholders in the consensus meeting. This would add *Termination of pregnancy.* |
| (3) Social determinants of health are not included in the core outcome set, e.g. homelessness, child sexual abuse, adverse childhood events, although acknowledged these are not illnesses. | We have considered social determinants as risk factors for developing the outcomes. |
| (4) Concerns that there are no long-term outcomes for children. | Some of the children’s core outcomes apply in the longer term, such as *Cerebral Palsy, Visual impairment, Needing complex care, Children’s mental health & behavioural disorder.* Other longer term outcomes that would manifest when the child is older, such as *Education attainment* and *Social participation* did not reach *Consensus in* in the Delphi surveys. |
| (5) Combining specific components of *Involvement in care decisions* (overall care, types of birth, pain relief), and perhaps with *quality & experience of care*. | Based on the group discussions, we will keep these outcomes separate. |
| (6) Concerns for including *Neurodevelopmental condition* in the core outcome set.  Including it in the core outcome set will encourage unethical studies that blame mother’s choices (pain relief, caesarean section, breastfeeding) and lead to mothers being denied certain mode of birth. This outcome is ableist and eugenics. | *Neurodevelopmental condition (children’s outcome)* reached *Consensus in* in the Delphi surveys. There is representation of women with neurodevelopmental condition in the Delphi surveys, consensus meeting and our Patient and Public Involvement advisory group.  Percentage of participants that rated this outcome as *Critically important* in the second Delphi*:*  - All participants 76.52%  - Women 76.92%  - Health professionals / researchers 76.32%  We will highlight the concerns in the manuscript. |
| (7) Concerns that there are very few experiences based outcomes in the core outcome set. | Experience based outcomes, such as *Birth experience* were included in the Delphi surveys, but did not reach *Consensus in.*  Percentage of participants that rated *Birth experience* as *Critically important* in the second Delphi*:*  - All participants 54.78%  - Women 64.10%  - Health professionals / researchers 50.00%  This does not mean this outcome is not important. We have discussed the importance of experience based outcomes in our focus group study that informed the design of our Delphi surveys. |

**First consensus meeting live poll for the 15 outcomes that were for *Further discussion***

**Criteria for inclusion:** ≥70% of all participants, or ≥70% of women stakeholders voting in favour of the outcome. These outcomes will be included in addition to the 45 outcomes that reached *Consensus in* in the second Delphi survey.

| **No** | **Outcome** | **2^nd^ Delphi survey** | | |  | **Consensus meeting final vote** | | | **Included** |
| --- | --- | --- | --- | --- | --- | --- | --- | --- | --- |
|  |  | **Yes, %** | | |  | **Yes, n (%)** | | |  |
|  |  | **All, n=116** | **Women / Partner, n=39** | **Clinicians / researchers, n=77** |  | **All, n=13** | **Women, n=6** | **Clinician/**  **researchers, n=7** |  |
|  | **Maternal outcomes** |  |  |  |  |  |  |  |  |
| 1 | Miscarriage | 68.14 | 73.68 | 65.33 |  | 12 (92.31) | 6 (100) | 6 (85.71) | Y |
| 2 | Termination of pregnancy | 63.39 | 71.05 | 59.46 |  | 8 (61.54) | 5 (83.33) | 3 (42.86) | Y |
| 3 | Gestational diabetes | 69.64 | 55.26 | 77.03 |  | 3 (23.08) | 0 (0) | 3 (42.86) | N |
| 4 | Gestational hypertension | 69.37 | 55.26 | 76.71 |  | 5 (38.46) | 1 (16.67) | 4 (57.14) | N |
| 5 | Involvement in care decisions for types of birth | 62.07 | 71.79 | 57.14 |  | 10 (76.92) | 3 (50.00) | 7 (100) | Y |
| 6 | Involvement in care decisions for pain relief | 63.79 | 71.79 | 59.74 |  | 7 (53.85) | 3 (50.00) | 4 (57.14) | N |
| 7 | Shoulder dystocia | 68.18 | 55.56 | 74.32 |  | 2 (15.38) | 0 (0) | 2 (28.57) | N |
| 8 | Quality & experience of care | 69.83 | 71.79 | 68.83 |  | 11 (84.62) | 4 (66.67) | 7 (100) | Y |
| 9 | Care for long-term conditions | 68.97 | 66.67 | 70.13 |  | 11 (84.62) | 6 (100) | 5 (71.43) | Y |
|  | **Children’s outcomes** |  |  |  |  |  |  |  |  |
| 10 | Separation of mother from baby | 66.38 | 74.36 | 62.34 |  | 10 (76.92) | 3 (50.00) | 7 (100) | Y |
| 11 | Blood transfusion (child) | 67.24 | 74.36 | 63.64 |  | 0 (0) | 0 (0) | 0 (0) | N |
| 12 | Inheritance of mother’s condition | 66.38 | 79.49 | 59.74 |  | 7 (53.85) | 4 (66.67) | 3 (42.86) | N |
| 13 | Quality of life (child) | 68.70 | 71.79 | 67.11 |  | 11 (84.62) | 4 (66.67) | 7 (100) | Y |
| 14 | General cognitive ability | 69.57 | 61.54 | 73.68 |  | 9 (69.23) | 3 (50.00) | 6 (85.71) | N |
| 15 | General gross motor ability | 67.54 | 56.41 | 73.33 |  | 4 (30.77) | 3 (50.00) | 1 (14.29) | N |
