# Supplementary material for: The development of a core outcome set for studies of pregnant women with multimorbidity
Source: BMC Med. 2023 Aug 21;21:314. doi: 10.1186/s12916-023-03013-3 (PMC10441728; doi:10.1186/s12916-023-03013-3)
Supplement: Supplementary file 7 — Additional file 7. Second consensus meeting report. [file 12916_2023_3013_MOESM7_ESM.docx]

## Additional File 7: Second consensus meeting report

**Second virtual consensus meeting: Core outcome set for studies of pregnant women with multiple long-term conditions (multimorbidity)**

**Date:** 24^th^ February 2023 **Time:** 1200pm to 1600pm UK time

**Aim of the meeting:** To reduce the 52 outcomes that have been included in the process so far (Delphi surveys and first consensus meeting) to a shorter list for the core outcome set.

***Premeeting preparation***

Premeeting was arranged with study participants to explain the aim of the meeting, explain what is a core outcome set, and explain the premeeting task. We provisionally aimed for 10 to 12 outcomes in the final core outcome set. We emphasised that just because an outcome is not included does not mean it is not important. Researchers studying specific research questions can still measure more specific and in depth outcomes related to the question.

***Premeeting task***

The premeeting task aims to help prepare participants for the discussion in the consensus meeting. Participants were invited to review the list of 52 outcomes, consider any overlaps in the outcomes and based on this which outcomes can be combined or removed from the core outcome set. Participants were also asked to consider which 5-8 outcomes they would choose to include. Participants were provided with plain English explanation of the outcomes, the results from the Delphi surveys and voting from the first consensus meeting; the overall results were presented, as well as stratified by stakeholder groups.

***Meeting structure***

One hour was dedicated to a **group discussion** on which outcomes can be combined or removed from the core outcome set, changes were made subsequently when there is no objections. Outcomes where decisions on combining or removing could not be made were kept for the next stage (voting). Participants were also asked if there were any outcomes that were removed from the discussion that they would like to add back to the voting stage.

This was followed by a **formal voting** for the remaining outcomes. Forty-five minutes were allocated for maternal outcomes and forty-five minutes for child outcomes. Participants were asked to do a binary vote for each of the remaining outcomes. The overall results and results stratified by stakeholder groups (people with lived experience, clinicians) were reviewed together. Any key areas where no outcomes were included, or outcomes where there were discrepancy between stakeholders (especially if an outcome was voted in by ≥80% women representative) would then be rediscussed and revoted (additional votes).

We also clarified that the meeting is to decide which outcomes to include. Decisions on how to define or measure the outcomes is beyond the scope of this meeting and current study, and would require a separate piece of work to reach consensus on.

***Criteria for inclusion in the second consensus meeting***

Outcomes that were voted in by ≥80% of all participants will be included in the final core outcome set. This was prespecified to all participants in the premeeting.

**52 outcomes included from the Delphi surveys and first consensus meeting**

| **Maternal outcomes** | **Children’s outcomes** |
| --- | --- |
| ***Survival***   1. Maternal death   ***Clinical: antenatal***   1. Miscarriage 2. Termination of pregnancy 3. Pre-eclampsia, eclampsia, HELLP syndrome 4. Placenta abruption 5. Placenta insufficiency 6. Venous thromboembolism   ***Clinical: peripartum***   1. Preterm premature rupture of membrane 2. Severe maternal morbidity 3. Postpartum haemorrhage 4. Hysterectomy 5. Maternal infection   ***Clinical: postpartum and longer term***   1. Development of new long-term conditions 2. Impact on long-term conditions   ***Resource use / care related outcomes***   1. Admission to intensive care unit 2. Involvement in care decisions (overall care) 3. Involvement in care decisions (types of birth) 4. Postpartum admission / readmission 5. Quality & experience of care 6. Care for long-term conditions   ***Mental health***   1. Suicide 2. Post-traumatic stress disorder 3. Perinatal mental health 4. Self-harm 5. Perinatal mental health support | ***Survival***   1. Death before birth (intrauterine death, stillbirth, perinatal death) 2. Death after birth (neonatal death, infant death)   ***Clinical: fetal***   1. Fetal growth restriction   ***Clinical: neonatal***   1. Gestational age at birth 2. Apgar score 3. Birth weight 4. Neonatal resuscitation required 5. Requiring intubation / ventilation 6. Neonatal birth injury 7. Neonatal sepsis 8. Brain injury on imaging 9. Neonatal respiratory distress syndrome 10. Necrotizing enterocolitis 11. Retinopathy of prematurity 12. Neonatal abstinence syndrome 13. Meconium aspiration syndrome 14. Separation of mother from baby   ***Clinical: infant***   1. Chronic lung disease / bronchopulmonary dysplasia   ***Clinical: longer term***   1. Congenital anomaly 2. Cerebral palsy 3. Children mental health & behavioural disorder 4. Need for complex care 5. Neurodevelopmental conditions   ***Life impact / functioning***   1. Visual impairment / blindness 2. Quality of life   ***Resource use***   1. Admission to neonatal unit (including intensive care) 2. Neonatal readmission to hospital |

**Voting results**

**First vote for maternal outcomes**

Three maternal outcomes were included in this round of voting:

1. Maternal death

2. Severe maternal morbidity

3. Change in existing long-term conditions

There was no discrepancy (an outcome not reaching the overall threshold, but one group voted ≥80% to include it) between stakeholder groups.

The voting results were reviewed when responses from 17 participants were received. Nine participants indicated they were clinicians and eight participants indicated they were women representatives. This mean one woman representative may have mistakenly voted as a clinician stakeholder (as there were nine women representatives and eight clinicians). No one identified themselves as voting twice. As the votes were anonymous, we were not able to rectify the error and presented the vote results as it is. This did not affect the overall score, but would affect the stakeholder breakdowns slightly.

**First vote for maternal outcomes**

| **No** | **Outcome** | **Percentage voting to include the outcome, n (%)** | | |
| --- | --- | --- | --- | --- |
|  |  | **All, n=17** | **Women, n=8** | **Clinician, n=9** |
| 1 | Maternal death | 17 / 17 (100) | 8 / 8 (100) | 9 / 9 (100) |
| 2 | Termination of pregnancy* | 8 / 16 (50) | 4 / 8 (50) | 4 / 8 (50) |
| 3 | Preterm premature rupture of membrane | 3 / 17 (18) | 2 / 8 (25) | 1 / 9 (11) |
| 4 | Severe maternal morbidity | 17 / 17 (100) | 8 / 8 (100) | 9 / 9 (100) |
| 5 | Hysterectomy | 4 / 17 (24) | 3 / 8 (38) | 1 / 9 (11) |
| 6 | Maternal infection | 7 / 17 (41) | 4 / 8 (50) | 3 / 9 (33) |
| 7 | Development of new long-term conditions | 12 / 17 (71) | 5 / 8 (63) | 7 / 9 (78) |
| 8 | Change in existing long-term conditions | 15 / 17 (88) | 7 / 8 (88) | 8 / 9 (89) |
| 9 | Involvement in care decisions (overall care) | 5 / 17 (29) | 4 / 8 (50) | 1 / 9 (11) |
| 10 | Involvement in care decision (types of birth) | 1 / 17 (6) | 1 / 8 (13) | 0 / 9 (0) |
| 11 | Postpartum admission / readmission | 8 / 17 (47) | 4 / 8 (50) | 4 / 9 (44) |
| 12 | Quality and experience of care | 10 / 17 (59) | 4 / 8 (50) | 6 / 9 (67) |
| 13 | Severe mental illness | 11 / 17 (65) | 5 / 8 (63) | 6 / 9 (67) |
| 14 | Development of new mental health conditions | 12 / 17 (71) | 6 / 8 (75) | 6 / 9 (67) |

*One participant did not vote for this outcome

**First vote for child outcomes**

Three child outcomes were included in this round of voting:

1. Survival of baby

2. Gestational age at birth

3. Neurodevelopmental conditions / impairment

There was discrepancy for ‘Separation of baby from mother’, with ≥80% women representative voting to include it.

**First vote for child outcomes**

| **No** | **Outcome** | **Percentage voting to include the outcome, n (%)** | | |
| --- | --- | --- | --- | --- |
|  |  | **All, n=17** | **Women, n=9** | **Clinician, n=8** |
| 1 | Survival of baby | 17 / 17 (100) | 9 / 9 (100) | 8 / 8 (100) |
| 2 | Gestational age at birth | 17 / 17 (100) | 9 / 9 (100) | 8 / 8 (100) |
| 3 | Birth weight | 13 / 17 (77) | 7 / 9 (78) | 6 / 8 (75) |
| 4 | Neonatal birth injury | 6 / 17 (35) | 5 / 9 (56) | 1 / 8 (13) |
| 5 | Neonatal sepsis | 8 / 17 (47) | 5 / 9 (56) | 3 / 8 (38) |
| 6 | Brain injury on imaging* | 6 / 16 (38) | 4 / 9 (44) | 2 / 7 (25) |
| 7 | Separation of baby from mother | 12 / 17 (71) | 8 / 9 (89) | 4 / 8 (50) |
| 8 | Congenital anomaly | 12 / 17 (71) | 6 / 9 (67) | 6 / 8 (75) |
| 9 | Neurodevelopmental conditions / impairment | 15 / 17 (88) | 8 / 9 (89) | 7 / 8 (88) |
| 10 | Children mental health & behavioural disorders | 7 / 17 (41) | 4 / 9 (44) | 3 / 8 (38) |
| 11 | Need for complex care | 10 / 17 (59) | 7 / 9 (78) | 3 / 8 (38) |
| 12 | Visual impairment / blindness | 4 / 17 (24) | 3 / 9 (33) | 1 / 8 (13) |
| 13 | Quality of life (child) | 13 / 17 (77) | 7 / 9 (78) | 6 / 8 (75) |
| 14 | Admission to neonatal unit (including intensive care) | 12 / 17 (71) | 7 / 9 (78) | 5 / 8 (63) |
| 15 | Neonatal readmission to hospital | 1 / 17 (6) | 1 / 9 (11) | 0 / 8 (0) |

*One participant did not vote for this outcome

**First revote (maternal outcomes)**

There were concerns that votes for the following outcomes were split due to the overlapping concepts, leading to none being included.

1. ‘Quality and experience of care’, ‘involvement in care decisions’

2. ‘Severe mental health conditions’ and ‘development of new mental health conditions’

There was no objection for combining the first set of outcomes as ‘quality and experience of care’ for the revote. There was no consensus on how to combine the second set of outcomes, so both were included for the revote.

***Results:*** ‘Severe mental health conditions’ was voted through. The remaining two outcomes did not reach the inclusion threshold overall, but was voted in by ≥80% of women representative. There was no objection to include ‘quality and experience of care’ in the core outcome set. ‘Development of new mental health conditions’ was put forward for the third revote.

**Second revote (child outcomes)**

Although not voted in overall, ‘separation of baby from mother’ was voted in by ≥80% of women representative in the first vote. There were concerns that votes for the following outcomes were split due to the overlapping concepts, leading to none being included.

‘Separation of baby from mother’ and ‘admission to neonatal unit’

However, no consensus was reached on how to combine these outcomes. Both were put forward for the second revote. Chair has asked that people vote for both if they felt both are important to avoid the splitting effect.

In the first vote, ‘quality of life (child)’ and ‘birth weight’ both received an overall 77% vote for inclusion, close to the inclusion threshold. Many outcomes (e.g. fetal growth restriction, placenta insufficiency) were removed with the understanding that they can be derived from gestational age and birth weight. ‘Quality of life (child)’ and ‘birth weight’ were put forward for revoting.

***Results:*** ‘Quality of life (child)’ and ‘birth weight’ were both voted through. ‘Separation of baby from mother’ and ‘admission to neonatal unit’ both did not meet the inclusion threshold.

**Third revote**

‘Development of new mental health conditions’ did not meet the threshold for inclusion in the first revote, but was voted in by ≥80% of women representative. ‘Separation of baby from mother’ and ‘admission to neonatal unit’ were combined and renamed as ‘separation of baby from mother for health care needs’ for the third revote.

***Results:*** ‘Development of new mental health conditions’ and ‘separation of baby from mother for health care needs’ were voted through.

**Fourth revote**

The core outcome set was reviewed by the wider research team and it was noted that the development of new or worsening of existing ‘severe mental health conditions’ overlapped with ‘change in existing long-term conditions (physical and mental)’ and ‘development of new mental health conditions’. This was also raised by a woman participant. Therefore all participants were contacted to vote on whether they agreed with removing ‘severe mental health conditions’ from the core outcome set.

***Results:*** ‘Severe mental health conditions’ was removed from the core outcome set, we emphasised that ‘change in existing long-term conditions’ should include both physical and mental health conditions.

**Revotes**

| **No** | **Outcome** | **Percentage voting to include the outcome, n (%)** | | |
| --- | --- | --- | --- | --- |
|  |  | **All** | **Women** | **Clinician** |
| **First revote (maternal outcomes)** | | | | |
| 1 | Quality and experience of care | 12 / 16 (75) | 7 / 8 (88) | 5 / 8 (63) |
| 2 | Severe mental health conditions | 13 / 16 (81) | 6 / 8 (75) | 7 / 8 (88) |
| 3 | Development of new mental health conditions | 11 / 16 (69) | 7 / 8 (88) | 4 / 8 (50) |
| **Second revote (child outcomes)** | | | | |
| 1 | Separation of baby from mother | 12 / 17 (71) | 7 / 9 (78) | 5 / 8 (63) |
| 2 | Admission to neonatal unit (including intensive care) | 13 / 17 (76) | 7 / 9 (78) | 6 / 8 (75) |
| 3 | Quality of life (child) | 14 / 17 (82) | 7 / 9 (78) | 7 / 8 (88) |
| 4 | Birth weight | 15 / 17 (88) | 8 / 9 (89) | 7 / 8 (88) |
| **Third revote** | | | | |
| 1 | Development of new mental health conditions | 14 / 17 (82) | 8 / 9 (89) | 6 / 8 (75) |
| 2 | Separation of baby from mother for health care needs | 15 / 17 (88) | 9 / 9 (100) | 6 / 8 (75) |
| **Fourth revote** | | | | |
| 1 | Remove *severe mental health condition* as overlaps with *change in existing long-term conditions (physical and mental)* and *development of new mental health conditions* | 16 / 17 (94) | 8 / 9 (89) | 8 / 8 (100) |

**Summary of group discussions**

| **General consideration for core outcome sets**  As we cannot have too many core outcomes, the core outcomes cannot be too specific.  Some outcomes have varying definitions (e.g. stillbirth, preterm birth) or have international variation (e.g. special baby care unit, different admission criteria). However, ‘gestational age at birth’, ‘birthweight’, can be easily measured by anyone in the world, and additional study specific outcomes can be derived from these. To consider hard outcomes if we are requiring everyone to measure the core outcomes.  Suggestion that we need to choose outcomes that can be identified universally by international classification of disease codes and to consider their availability in medical records as the aim is to make future studies homogenous and comparable in different countries.  There should be more outcomes for mothers, and child outcomes that are most important to the mother. Most research focuses on the baby, pregnant women may be counselled to accept certain intervention that health professionals felt to be in the best interest for the baby at the cost of the women’s quality of life. |
| --- |
| **Concerns on unethical research**  Women representative expressed concerns the core outcome set may be misused in unethical research, suggest we should make the effort when choosing and phrasing outcomes to avoid this. Clinical academic representative suggest this could be a separate discussion focusing on research ethics, use and misuse of outcomes. |
| **Maternal death**  *For:* As there is a lot of heterogeneity with multiple long-term conditions, maternal death would be a core outcome. Researchers would then be obliged to follow up study participants to check for survival at specific time points. Cause of death should then be captured with International Classification of Disease which can include any type of health conditions.  *Decision*: Kept for voting. |
| **Suicide**  *For:* Unsure if should be merged with ‘maternal death’ as it is the top cause of maternal death alongside cardiovascular disease. Could consider merging with ‘self-harm’.  *Against:* Captured by ‘maternal death’. Studies where the specific research question is perinatal mental health may measure suicide specifically and other perinatal mental health outcomes in detail. But should we require studies that are not related to perinatal mental health to always report suicide as an outcome?  *Decision*: Removed from core outcome set. |
| **Perinatal mental health**  Agreement to reduce the 5 perinatal mental health outcomes. The key is to capture whether there was perinatal mental health issues or not. To consider having proportionate number of perinatal mental health outcomes in the core outcome set as there are many other long-term conditions.  Suggestion of reducing this to 2 outcomes: severe mental illness and anther outcome for mental health conditions that are not severe mental illness, e.g. postnatal depression, anxiety, and other mild-moderate mental health conditions managed in primary care.  *Suggestion:* To use ‘perinatal mental health’ as an umbrella outcome.  *For:* Includes both development of new mental health conditions and relapse of existing mental health conditions.  *Against:* Important to differentiate between severity as ‘perinatal mental health conditions’ may be too broad and include a large number of women.  Important to distinguish the perinatal mental health conditions (new or aggravation of existing ones) attributable to the pregnancy, the multiple long-term conditions or interventions from those attributable to unrelated circumstantial events. Concerns that identifying mental health outcomes through diagnosis codes in medical records cannot distinguish this.  Discussed about addressing this by phrasing it as ‘new mental health conditions’. However, new mental health conditions that coincide with pregnancy does not mean pregnancy is the cause. This issue is addressed through study designs by having a comparator group, as this will take into account background events that happen by chance.  *Decision:* ‘Severe mental health conditions’ and ‘development of new mental health conditions’ were put through for the first vote. |
| **Severe mental health conditions, severe mental illness**  ***Two different approaches to ‘severe mental health conditions’***  There are two approaches to defining severe mental health conditions.  *First approach:* only includes a number of specific mental health conditions (e.g. bipolar disorder, schizophrenia), and is currently used in the UK and UK primary care records coding system.  *Second approach*: includes all possible mental health condition, and within each condition, only include those that are severe, measuring it on a severity scale using patient reported outcome tools (e.g. like rating pain on a scale of one to ten).  Some mental health researchers, especially those in perinatal psychiatry, do not want severe mental illness to only include long-term conditions that occur outside of the peripartum period. This is because this approach would not include acute but severe issues, such as acute trauma, which may or may not become post-traumatic stress disorder, according to how you are taken care of. If the second approach is taken, then severe trauma, complex trauma is a major outcome and it would be included in ‘severe mental health conditions’ as it is a mental illness and is included in the Diagnostic and Statistical Manual of Mental Disorders (DSM). Clinician representative suggests that we advocate for this definition in this current core outcome set work.  *For:* Recognised term, recognised composite outcome. Severe mental illness carries its own sets of risk and adverse outcomes. That does not mean other mental health outcomes are not important, the core outcome set would not stop other mental health outcomes being measured in studies where perinatal mental health is the focus of the research question.  *Against:* Concerns that in observational studies, confounding factors can lead to biases, socioeconomic factors may lead to different diagnosis rates in different groups. However this is a limitation with the study design, not with the choice of outcomes.  Concerns of study methodological limitations if diagnosis recorded in routine health records (primary care records or hospital records) are used to detect mental health outcomes. Limitations including misdiagnosis and subsequent misclassification of severity; does not provide information on the cause / triggers / associated significant life events; existing mental health conditions may be newly diagnosed around the time of pregnancy / birth due to the increased contact with health professionals. Concerns of stigma associated with severe mental illness.  Concerns that birth trauma / post-traumatic stress disorder will not be captured in ‘severe mental health conditions’, and researchers will continue to use the first definition, especially since there are variation of definitions used in different countries. Birth trauma / post-traumatic stress disorder also may not be reflected in ‘quality & experience of care’ (if good care was received) or ‘severe maternal morbidity’ (only capture physical trauma). This is an important outcome as it is common even in the general population as a result of difficult birth or negative care experience.  *Suggestions for revotes:* Initial suggestion for only this outcome to be revoted in the first revote, with the understanding that this includes both worsening of existing condition or a new condition.  *Decision:* As no maternal mental health outcome was voted in in the first vote, and no consensus was reached on how to combine the outcomes, both ‘severe mental health conditions’ and ‘development of new mental health condition’ was entered in the first revote. ‘Severe mental health conditions’ was voted in in the first revote, ‘development of new mental health conditions’ was voted in in the third revote.  However, in the final core outcome set, there was overlap between ‘severe mental health condition’ with ‘change in long-term condition’ (which includes mental health conditions) and ‘development of new mental health conditions’. Participants were contacted individually to ask if they agree with removing this outcome because of the duplication. |
| **Development of new mental health conditions**  *For:* This outcome was proposed because ‘change in long-term condition’ and ‘development of new long-term conditions’ would not cover new mental health conditions that are short-term, e.g. post-traumatic stress disorder that was managed in a timely manner. Could include the development of a new severe mental health condition.  *Suggestions for revotes:* To keep this in the revote option, as women representative wanted to be able to count minor mental health conditions. Concerned that birth trauma / post-traumatic stress disorder would not be captured by ‘severe mental health conditions’ if the definition with limited scope is being used. Birth trauma / post-traumatic stress disorder is common and seriously important for women, especially if they are as a result of care they received, or their physical conditions causing complication during birth.  *Decision:* Put forward for the first vote. As no maternal mental health outcome was voted in, this outcome was entered in the first revote. In the first revote, this outcome did not reach the 80% threshold, but was voted for inclusion by ≥80% women representative. Therefore it was entered in the third revote (second revote for this outcome) and was subsequently voted in. |
| **Maternal: miscarriage, termination of pregnancy**  **Child: death before birth, death after birth**  *Suggestions*: Combine ‘miscarriage’, ‘termination of pregnancy’, ‘death of baby before and after birth’ as ‘loss of baby’. Alternatively, combine as ‘pregnancy loss’ and to include ‘death before birth’, and keep ‘death after birth’ (perinatal / neonatal / infant) as a separate outcome. Reframe baby death / loss to ‘survival’ of child as that is what we really want to be looking at, there can be different definitions, e.g. survival at to a certain time frame, survival at 28 days (as opposed to neonatal death).  *For:* In all these circumstances the baby died, important to ensure the timing of when the death occurred is captured. Although miscarriage and termination of pregnancy is very different, both are pregnancy loss that may need additional support and postpartum care.  *Against:* Early pregnancy loss and losing baby at term feels different, the latter does not involve any decision making from the women, where else women may have been told to think about terminating their pregnancy because of their health. To keep ‘termination of pregnancy’ separate because in the first consensus meeting, there were discussions on the different reasons behind women choosing to have a termination of pregnancy, including reasons other than their health conditions, and whether they have been coerced by clinicians e.g. when the baby may have genetic conditions. The language ‘loss of baby’ may not be suitable to include ‘termination of pregnancy’.  *Decision:* Combined ‘miscarriage’, ‘death before and after birth’ (child) as ‘survival of baby’ and kept ‘termination of pregnancy’ separate for the vote. |
| **Pre-clampsia, eclampsia, HELLP syndrome**  **Placenta abruption**  **Placenta insufficiency**  **Postpartum haemorrhage**  **Admission to intensive care unit (maternal)**  **Severe maternal morbidity**  *Suggestions:* Remove some of the antenatal and peripartum complications that are already represented by ‘severe maternal morbidity’ (SMM).  *For:* SMM does not have a fixed definition, but the recognised definitions include the most severe manifestation of many pregnancy complications, e.g. heart stopping, blood clotting, or kidneys failing, which can happen in pre-eclampsia. Some of the antenatal complications (placenta abruption, placenta insufficiency, pre-eclampsia) are more specific to certain long-term conditions, and if we are looking for outcomes that are generically applicable to all types of long-term conditions, then the severe risk associated are captured by SMM.  *Decision:* Removed ‘pre-clampsia, eclampsia, HELLP syndrome’, ‘placenta abruption’, ‘placenta insufficiency’, ‘postpartum haemorrhage’, ‘admission to intensive care unit' (maternal). |
| **Hysterectomy**  Extremely rare, between 1 in 1000 to 1 in 1500 in the whole population. Peripartum hysterectomy is 0.3-0.4/1,000 births in UK. Women with multiple long-term conditions may be more at risk because they are more likely to have caesarean section, and may subsequently have a morbidly adherent placenta in a future pregnancy.  *For:* Should be a standalone outcomes as it is life changing  *Decision:* Kept in for voting. |
| **Preterm premature rupture of membrane**  *Against:* Covered by gestational age at birth (preterm birth). Currently listed under maternal outcomes, but potentially has a larger impact on the baby, as baby is then at risk of being born early or much higher risk of infection. Is it the process that matters or is it the actual outcome and impact on the baby? It is clearly distressing to have your water break early, but is one of the reason that it is distressing is because you know the impact this might have on your baby coming early and the problems that might entail?  *Decision:* Did not manage to confirm consensus for removing during the prevote group discussion, so this was kept in for voting. |
| **Quality and experience of care**  **Involvement in care decisions (overall care)**  **Involvement in care decisions (types of birth)**  *Suggestions:* To combine ‘involvement in care decisions’ under the umbrella ‘quality and experience of care’. ‘Involvement in care decisions (types of birth)’ is a subset of ‘involvement in care decisions (overall care)’.  *For:* If someone was very involved in their care decision, would that be broad enough to represent quality of care? Quality of care has so many aspect to it with involvement in care being one. Involvement in care decisions would influence experience of care. Quality of care can be measured with many scales, and usually includes involvement in care. Because there is a recognised framework for what is quality of care in maternity services, and that includes involvement in decision making, so we can confidently say the 2 involvement in care outcomes are included within quality of care. Mode of birth is an important care decisions but there are other important care decisions too.  *Against: ‘Quality and experience of care’* is too vague, more meaningful to keep involvement in care.  *Decision:* All three outcomes will be kept for the voting stage. In the revote stage, ‘involvement in care decisions’ were combined under ‘quality and experience of care’. |
| **Care for long-term conditions**  *Against:* This is a process measure, the way care is delivered, something that leads to an outcome. For instance, if the care for long-term condition is poor (process measure), it may lead to a change of status in the long-term condition or mental health (outcome).  *Decision:* Removed from core outcome set. |
| **Impact on long-term conditions**  **Development of new long-term conditions**  The wording for ‘impact on long-term conditions’ was not clear, whether this meant pathophysiological changes to the existing long-term conditions or the global holistic impact on the pregnant women and her care.  *Suggestion:* Combine these two outcomes. Rename as ‘change in long-term conditions’ to encompass worsening / improvement of existing conditions or an addition of new conditions.  *Against:* These two outcomes are very important but also are distinct entities.  *Decision:* These two outcomes were kept separate and both entered into the voting stage. ‘Impact on long-term conditions’ renamed as ‘change in long-term conditions’, and to mean worsening / improvement of existing physical or mental health conditions. |
| **Hospitalisation**  *Suggestion:* A proxy for severe / acute conditions that is either new onset or relapse of existing conditions.  *Against*: Some long-term conditions don’t lead to hospitalisation.  *Decision:* Not used as a replacement outcome for other outcomes. |
| **Gestational age at birth**  *For:* Keep this wording instead of changing to ‘preterm birth’ so it would include ‘post-term births’. ‘Gestation age at birth’ and ‘birth weight’ can be used to derived other outcomes.  *Decision:* Kept the wording unchanged and kept for the voting stage. |
| **Birth weight**  *For:* Important for deriving other outcomes such as fetal growth restriction, which can be derived from birth weight and gestational age. Also reflects the impact of maternal factors on baby, such as placenta insufficiency and hypertension in pregnancy. Acknowledge it is not perfect, but it is a good measure of how well the placenta has been able to support the developing fetus and to understand how well the baby has grown.  *Decision:* Kept in for the first vote and for the second revote. |
| **Fetal growth restriction**  *Against:* Can be captured by ‘birthweight’ and ‘gestational age at birth’.  *Decision:* Removed from core outcome set. |
| **Neonatal abstinence syndrome**  *Against:* Important to mothers who require specific medications during pregnancy, important in specific trials, e.g. trials looking at opiates, but may not be applicable to all trials and all long-term conditions.  *Decision:* Removed from core outcome set. |
| **Meconium aspiration syndrome**  *Against:* Very specific outcome. Would require resuscitation so could be combined with other conditions that requires resuscitation.  *Decision:* Removed from core outcome set. |
| **Necrotising enterocolitis**  **Retinopathy of prematurity**  **Neonatal respiratory distress syndrome**  **Chronic lung disease**  *Against:* These only apply to preterm babies and are also rare even in premature babies. ‘Gestational age at birth’ which covers preterm birth can be a proxy for these outcomes. They may also be overly specific.  *Decision:* Removed from core outcome set. |
| **Neonatal resuscitation required**  **Requiring intubation / ventilation**  *Against:* Is it the care / intervention that the baby receives that is important, or is it what happens to the baby in the end, i.e., the outcome of those intervention (e.g. admission to neonatal unit) that is important? Does it matter if there is no longer term impact on the baby, no neonatal unit admission, no separation from the mother?  *Decision:* Removed from core outcome set. |
| **Apgar score**  *Against:* Not particularly useful in premature babies. It is a snapshot of how the baby is at the particular point of time after birth. What is important is whether they go on to develop longer term problems like cerebral palsy or other conditions later on in life, or whether they are admitted to a neonatal unit or are separated from their mother. Significant difference in how babies are scored in different countries.  *Decision:* Removed from core outcome set. |
| **Congenital anomaly**  *Against:* This is an outcome of interest for specific maternal diseases or to specific drugs that may be teratogenic. For most studies on maternal chronic diseases, it could be dropped. Discussed whether this outcome can be combined with neurodevelopmental conditions if the impairment is not large.  *For:* There is a range of severity, for example lip defect can be very severe but can also be minor. Severity is subjective, how much a condition affects a child or a family cannot be easily judged often by the measures that we use. Mild congenital anomaly can still have long running consequences, such as relapse of the condition, needing surgical intervention when the child is older. Some women representative want to know whether taking medication during pregnancy can have an impact so this outcome is important.  *Decision:* Kept in for the voting stage. |
| **Children’s mental health and behavioural disorder**  *For:* Includes mental health conditions that occur in adulthood for the child. If risk of mental health conditions and behavioural disorder is higher in children born to mothers with multiple long-term conditions, then additional support may be needed.  *Against:* Questions on whether these are caused by the medications, the pregnancy or the environment the child is raised in, and these may not be life-long impairment, are short term or context specific. Concerns that there is high risk of conflation.  Women representative raised that it is not the behavioural disorder or a child that is agitated that is the actual issue, or impacting on the quality of life. It is a societal issue of parents or doctors wanting the child to behave the same (as the social norm). However, clinician representative says societal intolerance of children with behavioural disorder is beyond our control, and this can significantly impact on the way the child is taken care of, therefore it is an important outcome.  *Decision:* Kept in for the voting stage. |
| **Cerebral palsy**  *Suggestion:* Combine with ‘neurodevelopmental conditions’.  Clarified that ‘cerebral palsy’ was voted in in the Delphi surveys and was not discussed in the first consensus meeting (where outcomes that were borderline were discussed). At the survey design stage, this outcome was considered important enough to be a standalone outcome. Clarified that it is not specifically linked to any particular condition. It is often included as part of neurodevelopmental conditions (gross motor, fine motor, speech, vision, hearing etc).  *For:* Neurodevelopmental conditions, cerebral palsy, mental health and behavioural disorder makes a massive difference to patients and families as the children grow up. Patients and parents are less worried about the labels but more the impact on the family.  *Decision:* Combined with neurodevelopmental conditions. |
| **Neurodevelopmental conditions**  ***Against:***  *Concerns of underdiagnosis and misdiagnosis of autistic spectrum disorder*  Women representative noted that autistic spectrum disorder is not always diagnosed in childhood and diagnosis in women is often missed, therefore suggested to consider adding autistic spectrum disorder traits and behaviours to ‘Neurodevelopmental outcomes’.  Concerns that autistic spectrum disorder is often misdiagnosed as other behavioural or mental health conditions, concerns of the diagnosis being country and culture sensitive, rather than a clear criteria for more severe learning disability, genetic conditions, and physical impairment.  Concerns that undiagnosed autistic spectrum disorder in the mother may lead to misattribution of mother’s medication to children’s neurodevelopmental conditions, instead of attributing to genetic causes.  *Concerns of unethical research, stigma, eugenics, ableism*  The issue for autism is not the child’s behaviour, but society is not tolerant of the child’s behaviour. Concerns that researchers are conducting studies to proof certain interventions cause autistic spectrum disorder, which limit pregnant women’s access to certain interventions.  *Response:* Beyond the scope of the core outcome set work to fix the problems with all research, and would not be solved by not recording the outcome.  *Suggestion for renaming as ‘impairment’*  Could these outcomes be combined and be renamed as ‘impairment’ instead of listing it as neurodevelopmental conditions or mental health conditions, and researchers can specify which actual impairment is it impacting, e.g. visual impairment, speech impairment, learning disability, noise sensitivity. This would have more value, be less stigmatising and guard against eugenics, unethical research and misuse of the core outcome set, e.g. studies to link vaccine with autism. Discussed about challenges of protecting the core outcome set from being misused, difficulty with relying on researchers’ good intention when studying outcomes such as children mental health and learning disability.  On the spectrum of impairment, it is the severe end of ‘intellectual disability’ that is more important when considering the need for care, services and quality of life. Truly impairing learning disability is what should be kept instead of general conditions like autistic spectrum disorder or attention deficit hyperactive disorder that don’t always cause learning disability or significant impairment.  *Response:* How a condition impairs the child is quite subjective and difficult to measure, it depends on lots of factors, including the environmental the child lives in. A ‘disorder’ or ‘impairment’ may be too broad and may need more narrowing down. Important point on threshold on when some things become problematic and some things don’t, this has to be considered at a later stage in a separate work defining the outcomes.  There is a clear question of whether we have neurodevelopmental impairment as an overarching outcome, or we separate that down into the individual domains: such as motor impairment (e.g. cerebral palsy), hearing impairment, vision impairment, social and communication impairment (e.g. autism) and cognitive impairment. For example, in the neonatal core outcome set study, the research team break it down into the individual components, some were important enough to go through to the core outcome set, some were not. For this core outcome set, we could split these up too, but we are also trying not to lead to more outcomes. But for an area where this is so important, we could do that.  *Response:* Not asking for the impairment domains to be listed separately, but putting everything under the same umbrella, but change the wording of the outcome so it is not focusing on specific conditions but on the actual impairment.  ***For:***  *Importance of studying neurodevelopmental conditions*  The whole context of this work is multiple long-tern conditions in pregnancy. Many of these women take medicines that they cannot stop because of managing their multiple long-term conditions in pregnancy.  For example, for studies of women with epilepsy in pregnancy, if we had not included the concept of autistic spectrum disorder and attention deficit hyperactive disorder, we would never have discovered the problems associated with valproate and being able to relieve women of any anxiety for some of the newer anti-seizure medications that are not associated with these outcomes. Eugenics is a completely separate discussion to what we are having here.  We are not making value judgement on the outcome conditions that we are measuring. However if a medication would lead to more people having an outcome conditions, we would want to know that. People would want to have that information when they are making decisions for their own care, so recording it in research is a way of providing information to people who are faced with that decision in the future.  Need to capture developmental outcomes long-term up till adulthood of children whose mother have taken medication such as valproate for epilepsy or bipolar disorder. We need clear definition of what we mean by neurodevelopmental conditions, including intellectual disabilities, which is the major negative impact associated with or without autistic spectrum disorder, attention deficit hyperactive disorder, and other conditions.  *Decision:* Kept in for the voting stage. Important to include the ethics and importance of this outcome not being misused in the discussion in the manuscript. Clearly a lot of controversy, challenges in diagnosing these and variation between groups in society, different countries, different settings, which are all important factors to be considered in the next stage when determining how outcomes are defined. But the concept of neurodevelopmental outcomes is very important. |
| **Physical impairment (child)**  *Question:* Concerns there is no outcome for physical impairment for children.  *Response:* Difficult to understand how we would measure that. There is no good composite outcome like there is for maternal morbidity. A lot of the key physical impairment, such as cerebral palsy, are included in neurodevelopmental outcomes and these are often related to birth issues. So some of the child outcomes for physical impairment has been captured. Acknowledged that some other physical health conditions that might affect the child, for example asthma, are not included within neurodevelopmental outcomes, but birth factors are less commonly related to those outcomes, and they had not come through from previous phases of the core outcome set process, which makes it difficult to bring them in at this stage. |
| **Composite outcomes for babies**  *Question:* Is there an equivalent of ‘severe maternal morbidity’ for children?  *Response:* There is no equivalent of ‘severe maternal morbidity’ for children, or accepted common list of severe neonatal morbidity. There are various different combinations of complications that can occur in very premature babies but that is not really applicable to the wider population. |
| **Quality of life (child)**  *For:* An important child outcome, often separate from the condition / illness.  Need to consider measurability, very subjective. Should be a self-account of the child and where not possible, accounts of the parent. Research is getting better at measuring this outcome. It is still a challenge to measure it in very small babies, but that is not to say it is not important and doesn’t mean we should not use the core outcome set to push forward the agenda for people to develop a tool to measure it.  Quality of life (maternal) is raised as important too, but this did not make it through in the Delphi surveys.  *Decision:* Kept in for the voting stage. This outcome was put forward for a repeat vote as it was close to the inclusion threshold in the first vote for child outcomes. |
| **Birth injury**  *Further explanation:* An injury from the birth itself, commonly used to talk about conditions when the baby gets stuck and they have to be pulled out or delivered quite rapidly and often with quite a lot of force. They can get fractures of their arm or their shoulders, and can have injuries to their nerve in their arms. It could also include injuries related to babies being cut from a caesarean section. All these outcomes are rare. |
| **Neonatal sepsis**  *Further explanation:* A severe form of infection that is common in the neonatal period. Neonatologist spend a lot of time giving babies antibiotics to prevent this from happening. Affects preterm babies more but also affects term babies. |
| **Brain injury on imaging**  *Further explanation:* Imaging is something that most babies would not get routinely. Babies that were born preterm or go to neonatal unit will often, in a high income setting, get ultrasound scan/s of their brain. So this outcome is important to babies that go to the neonatal unit, but it is not so relevant to the wider group of babies that are not born preterm. Injury on imaging is only a proxy marker of the effect that it would have on the baby in the longer term, such as neurodevelopmental problems, blindness or need for complex care. |
| **Neonatal readmission to hospital**  *Further explanation:* ‘Neonatal readmission to hospital’ is when the baby have to come back into hospital in the first month after birth, after they have gone home or left the hospital. In contrast, ‘admission to neonatal unit’ would cover instances where a baby was admitted shortly after birth or in the next day or so when the mother is still in hospital, or being admitted during the initial stay.  ‘Neonatal readmission to hospital’ is not a universally accepted outcome and there are slight challenges with it. Where they go on readmission is very setting / country specific (e.g. paediatric ward, postnatal ward, neonatal unit). |
| **Postpartum admission / readmission (maternal)**  *Suggestion:* Combine with ‘admission to neonatal unit’ as ‘separation of baby from mother’ as women are stressed about not being able to look after their baby when these two situation occurs.  *Against:* Postpartum admission / readmission may not always mean separation of baby from the mother.  *Decision:* Kept in for the voting stage. |
| **Admission to neonatal unit**  In lots of settings around the world, there may not be access to neonatal units. Challenges with international variation on the wording used to describe babies who receive extra care. E.g., in the UK, some hospital have a unit that covers both Intensive and Special care (different levels) and it is not easy to differentiate which they have received.  To consider whether it is the admission to neonatal unit that is important, or is it the separation from mother to receive care somewhere else that is important, and whether it can be rephrase to incorporate that and be reflective of the wider world.  To consider putting a timeframe e.g. neonatal unit admission / additional medical care for more than 24 hours or 48 hours to account for variation in doctor’s experience and threshold for admission.  *Suggestion:* Combine as ‘separation of baby from mother’.  *Against:* Although this outcome overlaps with separation of baby from mother, women representative feel it is more than the separation. It comes with separate stress and aggravation related to the neonatal unit admission. Mothers are worried about the long-term consequences for the baby and the mother.  Separation of baby from mother may not capture the need for additional care for newborn baby in circumstances where kangaroo care (skin-to-skin care) is provided, e.g. for preterm and low birth weight baby in resource limited settings / lower middle income countries as an alternative. However this would be captured by ‘gestational age at birth / preterm birth.’  *Decision:* Kept in for the voting stage. Definition of this outcome (time frame, setting) is for discussion in a separate piece of work. |
| **Separation of baby from mother**  Current wording is vague as it includes both hospitalisation of the mother and hospitalisation of the baby. Discussed the need for defining the types of separation, for instance, differentiating between separation for a few minutes for a blood test as oppose to separation for a few weeks for admission to neonatal unit. The definitions and threshold setting is beyond the scope of this consensus meeting and would need to be addressed in a separate piece of work. Clarified the decision now is whether the idea that your baby is taken away or separated from you for medical reasons, should be included as a core outcome.  *For:* Very strong support from women representatives to keep this in, it comes with associated stress and anxiety. Both women and clinician felt the separation, the taking my baby away from me, regardless of the cause, is the issue.  ***Impact on feeding and bonding***  Women will be worried about whether they will be able to breastfeed or bond with the baby. Clinician representative felt this is an important proxy for infant feeding which was not voted in from the Delphi survey.  ***Impact on maternal mental health***  Women representative shared the anxiety they felt when their child was at risk of / has been admitted to neonatal unit, and how this was influenced by previous pregnancy events or influences whether they experience anxiety in future pregnancies. These examples illustrate the impact of the separation on maternal mental health.  *Suggestions for renaming:*   - ‘Separation of baby from mother in order to receive neonatal care’ if this was going to replace the neonatal admission outcome. - ‘Separation of baby from mother for health issues’ to rule out the temporary, less scary separation and keep the one where mother is more scared for the baby or her own health issues. - ‘Separation for infant or neonatal issues’ - ‘Separation of baby from mother for care / location of care delivery’, but is the latter the same as admission to neonatal unit? Neonatal unit may not be universal globally. - ‘Separation for baby reasons? For delivery of neonatal care?’ But these would not cover if mother was admitted to intensive care, which was covered with the broad separation of baby from mother - Women representative suggested ‘separation of baby from mother for health care needs’ so it would cover both the health needs for baby and mother.   *Decision:* Kept in for the first child outcomes voting, and for the revote. In the final revote, this was combined with ‘admission to neonatal unit’ and renamed as ‘separation of baby from mother for health care needs’. |
